# Mesoderm and myogenesis-related lncRNAs as Potential Markers of Myogenic Differentiation of Control and *miR145* or *miR181* Stimulated Mouse Pluripotent Stem Cells

**DOI:** 10.1007/s12015-025-11034-z

**Published:** 2025-12-17

**Authors:** Karolina Archacka, Anna Ostaszewska, Karolina Romanczuk, Anita Florkowska, Iwona Grabowska, Aleksandra Olszak, Joanna Molska, Maria A. Ciemerych

**Affiliations:** Department of Cytology, Institute of Developmental Biology and Biomedical Sciences, Faculty of Biology, University of Warsaw, Miecznikowa 1, Warsaw, 02-096 Poland

**Keywords:** Mouse, ESCs, Myogenic differentiation, MiRNAs, LncRNAs, MiR145, MiR181

## Abstract

Non-coding RNAs play a crucial role in the regulation of gene expression, impacting all processes occurring in a living cell. Among them are microRNAs (miRNAs) and long non-coding RNAs (lncRNAs), which interplay with each other modulating the expression of target genes. In the current study, we focused on lncRNAs described as involved in mesoderm specification or proliferation and differentiation of myogenic precursor cells and verified which of them may indeed be useful as candidate markers of mesodermal/myogenic differentiation of pluripotent stem cells (PSCs). As a model, we used two mouse embryonic stem cell (ESC) lines. We compared cells differentiating in the control medium with those induced to undergo myogenic differentiation by retinoic acid, insulin, transferrin, and selenium treatment. We showed that such induced ESC differentiation upregulated expression of few lncRNAs, i.e.,* yylncT*, *H19*, *SRA*, and *linc-YY1*, in both lines. The expression of other analyzed lncRNAs changed in the ESC line specific manner. Next, we studied the impact of *miR145* or *miR181*, which we have previously documented to elevate myogenic gene expression, on lncRNA levels. We found that *miR145* overexpression resulted in decrease in *H19* in both ESC types. Another common change observed in both cell lines was decrease of *H19* in response to *miR181*. Again, the expression of most lncRNAs known as mesodermal and myogenic regulators and studied by us occurred in ESC line-dependent, and diverse manner. For the first time we verified if and which lncRNAs may serve as a potential indicators of up-and-coming myogenic differentiation. However, it should obviously be tested by extensive studies that include multiple different PSC lines.

**Supplementary Information:**

The online version contains supplementary material available at 10.1007/s12015-025-11034-z.

## Introduction

The knowledge accumulated since the derivation of the first embryonic stem cell (ESC) lines [1] secures a background for studies on pluripotent stem cell (PSC) self-renewal and differentiation. The availability and detailed information about PSC differentiation methods is crucial, as these cells are widely considered to be used in regenerative medicine or tissue engineering. Research focusing on the myogenic differentiation of PSCs may take advantage of either overexpression of selected genes or the design of special culture media and conditions. Overexpression of crucial regulators of skeletal muscle development, e.g.,* Pax3* or *Pax7* transcription factors, allows efficient turning of PSCs into myogenic precursor cells (MPCs) (e.g, [2, 3]). In addition, non-invasive methods that facilitate PSC myogenic differentiation by supplementing culture media with growth factors or cytokines (summarized in [4–7]), or by environment mimicking embryonic development, were published [6, 8]. However, the majority of these methods are multistep, time-consuming, and for this reason hardly feasible to use in clinics. We previously showed that overexpression of miRNAs regulating pluripotency or myogenesis, such as *miR181* or *miR145*, can serve as a trigger changing the fate of differentiating ESCs [9].

*miR145* has been shown to downregulate pluripotency factors - OCT4, SOX2, KLF4 [10, 11], as well as to induce the expression of mesodermal markers, such as Nodal (e.g, [12]), to affect WNT pathway [13], and to suppress osteogenic and promote myogenic differentiation of C2C12 myoblasts [13, 14]. *miR181* homolog levels have been shown to increase during myogenesis [15] and have a positive effect on myogenic differentiation in mammals [16, 17], birds [18], or fish [19] (for a review, see [20]). During myogenic differentiation, it targets mRNA encoding the homeobox protein HOX-A11, that is, the repressor of terminal differentiation [21], as well as MYOSTATIN B [19] or YAP1 [17]. *Mir181* action may be blocked by long noncoding RNA (lncRNA) - *lncDLEU2* leading to inhibition of myogenic differentiation [22]. Moreover, *miR181* is known to block the translation of NAD-dependent protein deacetylase (SIRT1), involved in ESC differentiation in response to RA [23, 24].

In our previous work, by overexpressing *miR145*, we achieved induction of *Pax3* and *Pax7* in mouse ESCs [9]. We also observed an increase in the expression of the *Myod* transcript. Next, *miR181* increased the expression of myogenic markers, such as *Myod* and myosin heavy chain isoform 2 – *Myhc2*. It also significantly increased the number of cells containing the MYF5 protein. Importantly, using each of these miRNAs we were able to obtain myoblasts and, in case of *miR181* overexpression, myotubes with visible sarcomeric organization. We also showed that in differentiating ESCs, *miR181* upregulated the expression of many miRNAs - characteristic for mesoderm, MPCs, or skeletal muscle fibers [9]. However, we did not have any information about potential changes in other non-conding RNAs, i.e., lncRNAs. Thus, in the current study, we focused on lncRNA landscape in mouse ESCs undergoing myogenic differentiation in vitro, and potential linkage between lncRNAs and *miR145* or *miR181* used for ESC treatment. We followed lncRNAs that were already described as involved in various steps of mesoderm formation and myogenic differentiation.

Among them is *yylncT*, located within *T* locus encoding the BRACHYURY, the crucial mesoderm differentiation regulator [25]. Mesoderm formation is also under the control of *Mrhl* (meiotic recombination hotspot locus) [26]. *Malat1* (metastasis-associated lung adenocarcinoma transcript 1), which is transcribed from the same gene as *mascRNA*, is involved in the early stage of myogenesis - it promotes myoblast proliferation and prevents myogenic differentiation. Induction of myogenic differentiation, accompanied by *miR181* expression, leads to *Malat1* degradation [27]. *MascRNA*,* Sirt1AS*, and *Neat1v1* (nuclear paraspeckle assembly transcript 1) also support myogenic cell proliferation [28–33]. One of the first lncRNAs described as involved in the regulation of myogenesis – *H19* [34, 35], initially expressed in various tissues of the developing embryo, after birth is maintained in skeletal muscles only [34]. Its expression is initiated by MYOD and is elevated during both myoblast differentiation as well as skeletal muscle regeneration [36]. The silencing of *H19* leads to a decrease in MYOGENIN and MyHC levels. The MYOD axis is also regulated by *SRA* (steroid receptor activator) and *lncMyoD* which bind to MYOD and also promote the activation of other muscle specific genes [37, 38]. *LncMyoD* knockdown results in the inhibition of terminal muscle differentiation, mainly due to the failure to exit the cell cycle. Another lncRNA known to be engaged in myogenesis is *Linc-MD1* which is detectable in myogenic cells which start to differentiate and disappears at the myotube and myofiber stage [39–41]. *Linc-MD1* enables the translation of MAML1 (mastermind-like protein 1) and MEF2C (myocyte enhancer factor 2 C) which next induce the expression of muscle specific genes, e.g., *Myog* and *Myhc* [41]. The final stages of myogenic differentiation, i.e., formation of fully differentiated myotubes are regulated, e.g., by *Linc-YY1* [42, 43] and *ppp1r1b* which both act via regulation of PRC2 function resulting in upregulation of such genes as *MYOD*, *MYOG*, and *TBX5* [44]. Finally, *MEG3* is another lncRNA important for myotube formation - its silencing leads to the significant limitation of this process, as well as a decrease in myoblast proliferation and *Myod*, *Myog*, *Mef2*, and *Mck* expression [45, 46].

In the current study, we followed the expression of abovementioned lncRNAs, described as involved in mesoderm and myogenesis regulation: *H19*, *Linc-MD1*,* Linc-YY1*,* lncMyoD*,* Malat1*,* mascRNA*,* MEG3*,* Mrhl*,* Neat1v1*,* ppp1r1b*,* Sirt1AS*,* SRA*, and *yylncT* (listed in Table S1 which summarizes their main function and includes representative publications). We followed their pattern in differentiating mouse ESCs, control or treated with RA and ITS, used by us in previous studies to enhance mesodermal and myogenic differentiation of ESCs [9, 47, 48]. Next, we checked if and how transient overexpression of either *miR145* or *miR181* influences the expression of the lncRNAs mentioned above, for the first time showing the dynamic changes of these 13 lncRNAs in two ESC lines, and discovering cell line specific, different lncRNA patterns in response to *miR145* or *miR181* treatment. Our results allow us to verify if and which lncRNAs may serve as a potential indicator of PSC myogenic differentiation.

## Materials and Methods

### ESC Culture

Cell lines used in this project were mouse ESC lines: H2B-GFP obtained by Anna-Katerina Hadjantonakis and Virginia E Papaioannou by generating histone H2B-GFP fusion [49] or 7AC5-YFP derived by Andras Nagy and Anna-Katerina Hadjantonakis by random integration of eYFP (ATCC). ESCs were cultured under previously described standard conditions (e.g, [9, 47, 50]). The medium for ESC culture (ESC medium) was KnockOut Dulbecco’s Modified Eagle’s Medium (KnockOut DMEM, Gibco) supplemented with 15% heat-inactivated fetal bovine serum (FBS; Performance Plus, Gibco) with the addition of non-essential amino acids (0.1 mM, Gibco), L-glutamine (2 mM, Gibco), β-mercaptoethanol (0.1 mM, Sigma-Aldrich), penicillin and streptomycin (5000 units/ml each, Gibco), and murine leukemia inhibitory factor (LIF, 1000 IU/ml, ESGRO, Chemicon International). The medium was changed daily and the ESCs were passaged every 3 days onto mouse MEFs derived by us previously, for other study, and inactivated according to Hogan et al. [9, 51]. Briefly, MEFs were maintained in DMEM (4.5 g/L glucose, Gibco) supplemented with 10% heat-inactivated fetal bovine serum (FBS, Gibco) and 5,000 U/ml penicillin–streptomycin (Gibco). Upon reaching confluence, MEFs were growth-arrested by treatment with mitomycin C (10 µg/ml, Sigma–Aldrich) for 2 h. The inactivated feeder cells were frozen for storage and thawed/plated one day before ESC seeding. Feeder layers were prepared by plating 2 × 10⁶ MEFs onto 100-mm culture dishes (4.5 × 10⁴ cells/cm²) pre-coated with 1% gelatin in PBS. After 24 h, 1 × 10⁶ ESCs were seeded onto the MEF layer per dish.

### ESC Differentiation

Before the differentiation procedure, ESCs and MEF feeder layer were incubated in 0.05% trypsin-EDTA in PBS (Gibco) for approximately 2 min. The cells were suspended in ESC medium, seeded twice onto 1% gelatin-coated culture plates, and incubated at 37°C for 20 min. Such treatment allowed MEFs to adhere to the plate and left ESCs floating within the culture medium. ESCs were collected and passaged to another gelatin-covered culture plate, and used for further analyses. ESC differentiation, that is, embryoid body (EB) and embryoid body outgrowth (EBO) formation, was performed according to our previously used protocol involving treatment with RA and ITS [9, 47]. Briefly, 800 ESCs were suspended in 30 µl of ESC medium that lacked LIF. Drops of medium containing ESCs were placed on covers of culture plates that were filled with phosphate-buffered saline (PBS) and cultured at 37°C allowing for EB formation. On day 2 of culture in hanging drops, the EBs were transferred to low-adhesive plates filled with ESC medium that lacked LIF and further cultured in suspension. On day 7 of culture, EBs were transferred onto gelatin-coated coverslips placed in 6-well culture plates to allow EBO formation. Cells were cultured under control or differentiation-inducing conditions, as previously described [9]. Differentiation-inducing cultures were carried out in DMEM lacking LIF and supplemented with 15% FBS from day 0 until day 10. From day 10 until day 13, cells were cultured in DMEM supplemented with 20% FBS. Next, the concentration of FBS was reduced to 10% (at day 13) and 5% (at day 14). Subsequently, FBS was withdrawn and the culture medium was composed of DMEM and F12 (1:1, Gibco) supplemented with 1% N2 (Gibco). Between days 2 and 7, the medium was supplemented with retinoic acid (RA, 30 nM in DMSO, Sigma–Aldrich) and insulin, transferrin, selenium (ITS, 1%, Gibco). Culture media was changed every 2 days. Cells were analyzed on day 0 (control ESCs), 7 (EBs), 14 (EBOs), and 21 (EBOs) of the culture.

### Differentiating Cell Microscope Examination

To monitor progression of ESC myogenic differentiation EBOs, cultured as described above, were examined and photographed using a Nikon eclipse TE200 microscope and a Nikon Digital Sight DS-U2 camera. Next, EBOs cultured for 21 days were fixed with 4% PFA in PBS at room temperature for 10 min and then permeabilized with 0.5% Triton-X 100 (Sigma-Aldrich) in PBS at room temperature for 5 min. Nonspecific antibody binding was blocked by incubation of cells in 3% bovine serum albumin (BSA, Sigma–Aldrich) in PBS at room temperature. The EBOs were incubated with anti-Myosin (skeletal) antibody produced in rabbit (Sigma-Aldrich, M7523) diluted 1:200 or troponin T antibody produced in mouse (Abcam, ab8295) diluted 1:100 in 0.5% BSA in PBS, in 4°C, overnight. The samples were incubated in the solution of the appropriate secondary antibody conjugated with Alexa 488 or Alexa 594 (Thermo Fisher Scientific) diluted 1:200 in 0.5% BSA in PBS, at room temperature, for 2 h. The nuclei were visualized by incubation of the sample in Hoechst 33,342 (Sigma) diluted 1:1000 in PBS for 5 min. Finally, the samples were mounted with a fluorescent mounting medium (DakoCytomation). The samples were analyzed using the Axio Observer Z1 confocal microscope (Zeiss, Carl Zeiss Inc., Jena, Germany) equipped with the LSM 700 application software (Carl Zeiss Inc., Jena, Germany). For each analysis, three independent experiments were performed.

### Mimic miRNA Transfection

Mimic *miR145* or *miR181* (Thermo Scientific) were introduced into ESCs using X-tremeGENE siRNA Transfection Reagent (Roche), as previously described [9]. Briefly, ESCs (3 × 10^4^) were seeded in gelatin-coated 6-well plates in ESC medium containing LIF. The next day, the ESCs were transfected with Xtreme Gene siRNA Transfection Reagent (Roche), according to the manufacturer’s protocol with either synthetic mmu-*miR145*a-5p (50 nM) or mmu-*miR181*a-5p (50 nM). Control cells were treated only with transfection reagent (MOCK). The efficiency of transfection was verified by transfecting ESCs with 50 nM Alexa Fluor 647 conjugated mimic miRNA (mirVana miRNA Mimic, Negative Control #1, Alexa Fluor 647 labeled - Dy647; Thermo Fisher) and 48 h later assessing the proportion of cells transfected with flow cytometer CytoFlex (Beckman) equipped with CytExpert software. Cells were first gated using FSC/SSC parameters, followed by doublet exclusion based on FSC-H/FSC-W. Subsequently, fluorescence of the cell population was evaluated in the APC-A channel (excitation using a 633 nm red laser, detection using a 670/30 bandpass filter). Background fluorescence was determined using the negative control (MOCK – cells treated only with the transfection reagent). Each analysis was performed in three independent experiments, always including negative and control samples.

### C2C12 Culture

Proliferating C2C12 cells were cultured in DMEM with high glucose (4.5 g/l, Gibco) supplemented with 10% FBS (Gibco) and antibiotics (Gibco) for 4 days and then collected. To induce differentiation, the medium was changed to DMEM supplemented with 2% horse serum (HS, Gibco) and antibiotics at day 4 of the culture. After additional 2 days of culture differentiating C2C12 were collected and frozen in −80°C until further analysis.

### Co-culture of ESCs and C2C12 Myoblasts and Fusion Index Assessment

C2C12 cells were also used for the co-culture with ESCs transfected with *miR145* or *miR181*. Forty eight hours after transfection ESCs were seeded on the layer of C2C12, previously cultured in DMEM supplemented with high glucose (4.5 g/l, Gibco), 10% FBS (Gibco) and antibiotics (Gibco), for 2 days. In control variants, differentiating myoblasts were co-cultured with control, MOCK ESCs. Co-cultures were performed under standard conditions: 37°C, 5% CO_2_ for one week. Next, cells were fixed with 3% PFA, rinsed with PBS, and stained with May–Grünwald and Giemsa, according to manufacturer’s protocol (Sigma-Aldrich). Stained cells were used to assess the fusion index serving as a parameter of functional myogenic differentiation outcome. Images were captured with an inverted light microscope (Nikon Eclipse TE200, 20X objective) in three random fields per well. The fusion index was calculated as the ratio of nuclei within myotubes to the total number of nuclei, and presented as a percentage.

### qRT-PCR

mRNA and lncRNA were isolated from E13.5 mouse embryos obtained from F1(C57Bl6NxCBA/H) females mated by males of the same cross, mouse C2C12 cells, mouse ESCs, EBs, and EBOs, both control ones and transfected with mimic miRNAs using mirVana miRNA (Thermo Fisher) or High Pure miRNA Isolation Kit (Roche). Next, the samples were treated with Ambion^®^ TURBO DNA-free™ kit. The purity and concentration of RNA were evaluated by the NanoDrop TM One Spectrophotometer, Thermo Scientific™. Then, for gene analysis, cDNA was synthesized using the RevertAid First Strand cDNA Synthesis Kit (Thermo Fisher), according to the manufacturer’s instructions. mRNA expression was analyzed by quantitative RT-PCR using mRNA TaqMan^®^ Gene Expression Master Mix (Thermo Fisher) and appropriate probes (details are summarized in Table S12), as described [9]. Expression of the following transcripts was analyzed: *T*,* Pdgfra*,* Kdr*,* Myf5*,* Myhc3*,* Myhc7*,* Myhc2*. The reference gene, *Actb* was selected for mRNA analysis on the basis of the initial experiment results. In this initial analysis we have used a few reference genes (*RNU43*, *U6B*, * 18S*, *Gapdh*, *Actb*) reported in the literature or included in the RA930A-1 LncRNA Profiler RT-qPCR Array Kit (System Biosciences) described further and used for lncRNA analysis. Based on the obtained results (best stability and reproducibility across different sample types analyzed) we have chosen *18S* as the best reference gene for lncRNA analysis and – as mentioned above - *Actb* as the best reference gene for mRNA analysis.

lncRNAs were analyzed using the RA930A-1 LncRNA Profiler RT-qPCR Array Kit (System Biosciences) or Poly(A) Polymerase Tailing Kit (Biosearch Technologies) followed by the RevertAid First Strand cDNA Synthesis Kit (Thermo Fisher), accordingly to the manufacturer’s protocols. Experiments were performed with 0.5–1 µg RNA. Specific primers for lncRNAs included in the RA930A-1 LncRNA Profiler RT-qPCR Array Kit (System Biosciences) or listed in Table S12 as well as Maxima SYBR Green/ROX qPCR Master Mix (Thermo Fisher) were used for analysis in Roche Light Cycler 96 using the following program: (a) 2 min − 50°C; (b) 10 min − 95°C; (c) 15 s − 95°C (50 cycles), followed by (d) 1 min − 60°C for lncRNAs analyzed using the RA930A-1 LncRNA Profiler RT-qPCR Array Kit (System Biosciences) or (e) 30 s – selected temperature (Table S12) and (f) 30 s − 72°C for other experiments. Data were analyzed using StepOne Software (Applied Biosystems).

### Statistical Analysis

Comparisons between experimental groups were performed using One-way ANOVA or Kruskal-Wallis test, depending on meeting the criteria of statistical tests. Statistical analysis was performed using GraphPad Prism 9 software. In each analysis, at least 3 independent biological samples were used.

## Results

### LncRNA Expression in Mouse Embryos and C2C12 Cells

**Fig. 1:**
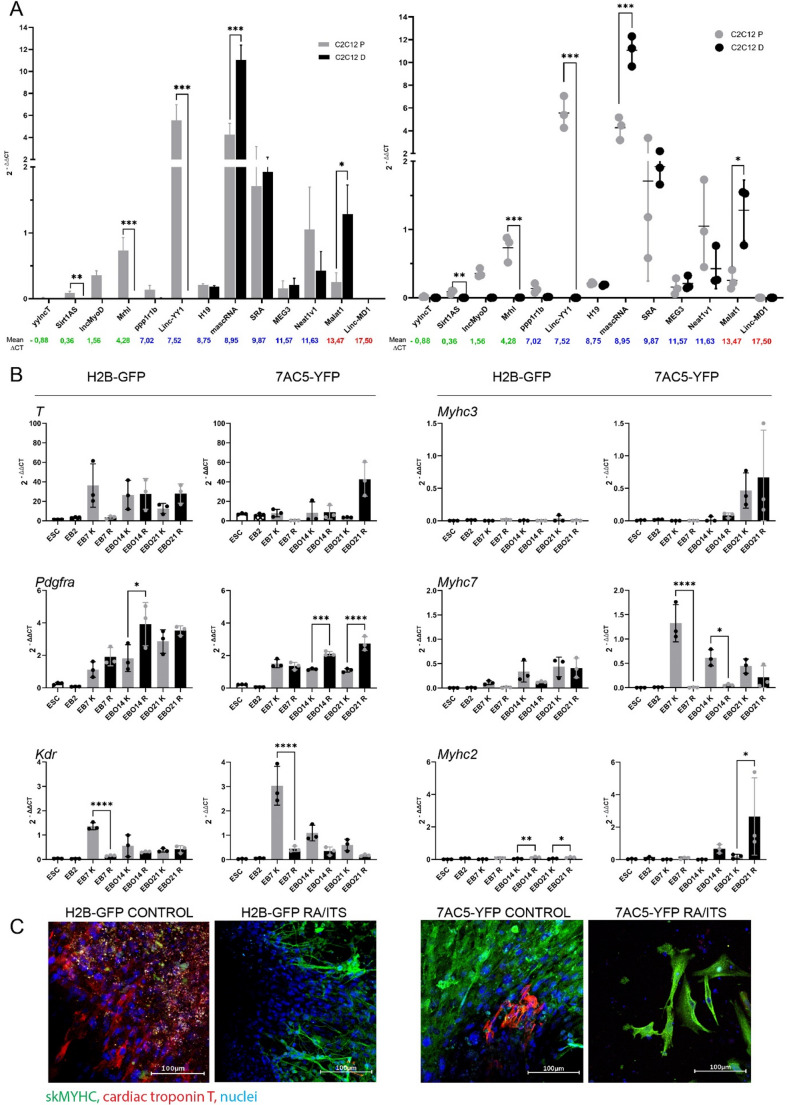
(**A**) Expression of selected lncRNAs in proliferating and differentiating C2C12 cells; (**B**) Expression of selected mesoderm and myogenic markers in H2B-GFP and 7AC5-YFP ESCs analyzed at the indicated time points. (**C**) Immunocytochemistry analysis of cardiomyocyte and skeletal muscle cell markers in EBOs generated in RA/ITS supplemented or control medium. (**A**) C2C12 P refers to proliferating C2C12 cells. C2C12 D refers to differentiating C2C12 cells. The numbers in color indicate the mean ΔCT for the mouse embryos. The mean ΔCT with standard deviation for C2C12 cells is shown on the left graph while values obtained for each biological replicate are shown on the right graph. The stars show significant differences between C2C12 P and C2C12 D in the level of expression of the same lncRNA. (**B**) K refers to cells cultured in control medium (grey columns). R refers to cells cultured in RA/ITS medium (black columns). Empty boxes indicate undifferentiated ESCs. Mean with standard deviation as well as values obtained for each biological replicate are shown. Stars show significant differences between cells cultured in different media (control vs RA/ITS) and analyzed at the same time-point. *p<0.05, **p<0.01, ***p<0.001,****p<0.0001. Each experiment was performed in 3 replicates. (**C**) Localization of skMYHC (green), cardiac troponin T (red), and nuclei (blue) in EBOs (21 day of culture) formed in control or RA/ITS-supplemented medium. Bar -100 µm.

First, we established the expression of selected 13 lncRNAs, related to mesoderm formation and myogenesis, using material isolated from the E13.5 mouse embryo, as well as control proliferating (P) and differentiating (D) mouse C2C12 cells serving as a model system to follow *in vitro* myogenic differentiation (e.g, [33, 44, 52]). E13.5 mouse embryo lysates provides suitable material to analyze the expression of various factors involved in the regulation of different tissues and organ development, including skeletal muscles (e.g, [9, 47]). RNA isolated from mouse embryo and C2C12 cells allowed us to verify the detectability of 13 selected lncRNAs: *yylncT*,* Sirt1AS*,* lncMyoD*,* Mrhl*,* ppp1r1b*,* Linc-YY1*,* H19*,* mascRNA*,* SRA*,* MEG3*,* Neat1v1*,* Malat1*, and *Linc-MD1* (Fig. 1A). Calculated ΔCT from 3 independent experiments, ΔCT mean values and standard error of the mean are presented in Tables S2 and S3. We showed that indicated lncRNAs were detectable in E13.5 embryos and some of them in proliferating or differentiating C2C12 cells (Fig. 1A, Tables S2, S3). Only the levels of *Linc-YY1* and *mascRNA* were higher in C2C12 cells, compared to the embryo.

### Myogenic Markers and lncRNA Expression in Differentiating ESCs – Impact of Culture Conditions

In our previous studies we compared the myogenic differentiation of a model ESC line, that is, D3 [53] and the one derived by us, i.e., B8 [47, 48]. We showed that RA and ITS treatment increases the expression of myogenic markers, such as *Myf5*, *Myod*, and *miR206* in EBOs at 21 day of differentiation [9]. In the current study we used 2 fluorescently labelled ESC lines - H2B-GFP and 7AC5-YFP ESCs - as this study was a part of the bigger project in which we characterized the impact of miRNAs at ESC differentiation both in vitro and in vivo. In in vivo part miRNA treated ESCs were transplanted into injured and regenerating muscles and the presence of GFP or YFP allowed us to follow the fate of these cells (Archacka et al., submitted). Many lines of evidence document that PSC lines may significantly differ in their sensitivity to culture conditions and ability to differentiate (e.g, [54, 55]). Thus, we first confirmed that H2B-GFP and 7AC5-YFP ESCs could be induced to differentiate using the RA/ITS-based protocol which we employed in the previous studies [9, 47]. We followed the expression of *T*,* Pdgfra*,* Kdr*,* Myhc3*,* Myhc7*, and *Myhc2* in ESCs, EBs (day 7 of differentiation), and EBOs (days 14 and 21 of differentiation) cultured either in control or RA/ITS supplemented medium (Fig. 1B). The expression of a primitive streak markers, i.e.,* T*, and then *Pdgfra* and *Kdr* genes characterize the mesoderm precursors that give rise either to myogenic or cardiomyogenic lineages, respectively [56]. In analyzed ESC lines we have not found any significant differences in *T* expression between cells cultured in different media while the levels of *Pdgfra* transcripts increased in both control and treated ESCs, but this increase was significantly higher in cells differentiating in the RA/ITS presence. Control culture conditions led to the up-regulation of *Kdr* expression, while RA/ITS medium did not have such an effect (Fig. 1B). This means that RA/ITS treatment ensured myogenic rather than cardiomyogenic differentiation as judged on the basis of *Pdgfrα* upregulation and *Kdr* downregulation in both cell lines. Analysis of *Myhc3*, that is, the embryonic form of myosin heavy chains did not document any significant changes, regardless of the cell line analyzed and the conditions used (Fig. 1B). Interestingly, in EBs and EBOs generated from 7AC5-YFP ESCs, the expression of other myosin heavy chains - *Myhc7*, characteristic for muscle fibers at earlier stages of maturation, was higher in cells cultured in control medium, while the level of *Myhc2*, characteristic of more advanced stages of fiber maturation, was higher in cells cultured in RA/ITS medium. The H2B-GFP ESCs did not show such differences in *Myhc7* expression but on day 14 and 21 cell cultured in the presence of RA/ITS upregulated *Myhc2* (Fig. 1B). Figure 1B presents significant differences only between cells cultured under different conditions (control versus RA/ITS) and analyzed in the same time point. Complete statistical results of indicated gene expression at different time points of the culture are shown in Figures S1 and S2. To verify differences revealed by RT-qPCR between cells differentiating in control and RA/ITS supplemented media, we analyzed EBOs fixed at day 21 of the culture and immunostained for cardiac troponin T and skMYHC, markers of cardiomyocytes and skeletal muscles (myoblasts), respectively. In accordance with RT-qPCR results described above, EBOs cultured in control medium were characterized by the presence of numerous cells positive for cardiac troponin T which were virtually absent in EBOs generated in RA/ITS supplemented medium (Fig. 1C). In turn, cells positively stained with anti-skMYHC antibody were more abundant in EBOs formed in differentiating medium than in control one (Fig. 1C). Differences in myogenic cell formation frequency were also supported by the results of light microscope analysis which revealed the presence of skeletal muscle cells with proper morphology in both types of EBOs, however, they were more noticeable in EBOs generated in RA/ITS supplemented medium (Fig. S3A). Such EBOs were also characterized by significantly higher level of *Myf5* transcript in comparison to control EBOs at day 21 (Fig. S3B). All indicated observations were common for both analyzed ESC cell lines.

**Fig. 2:**
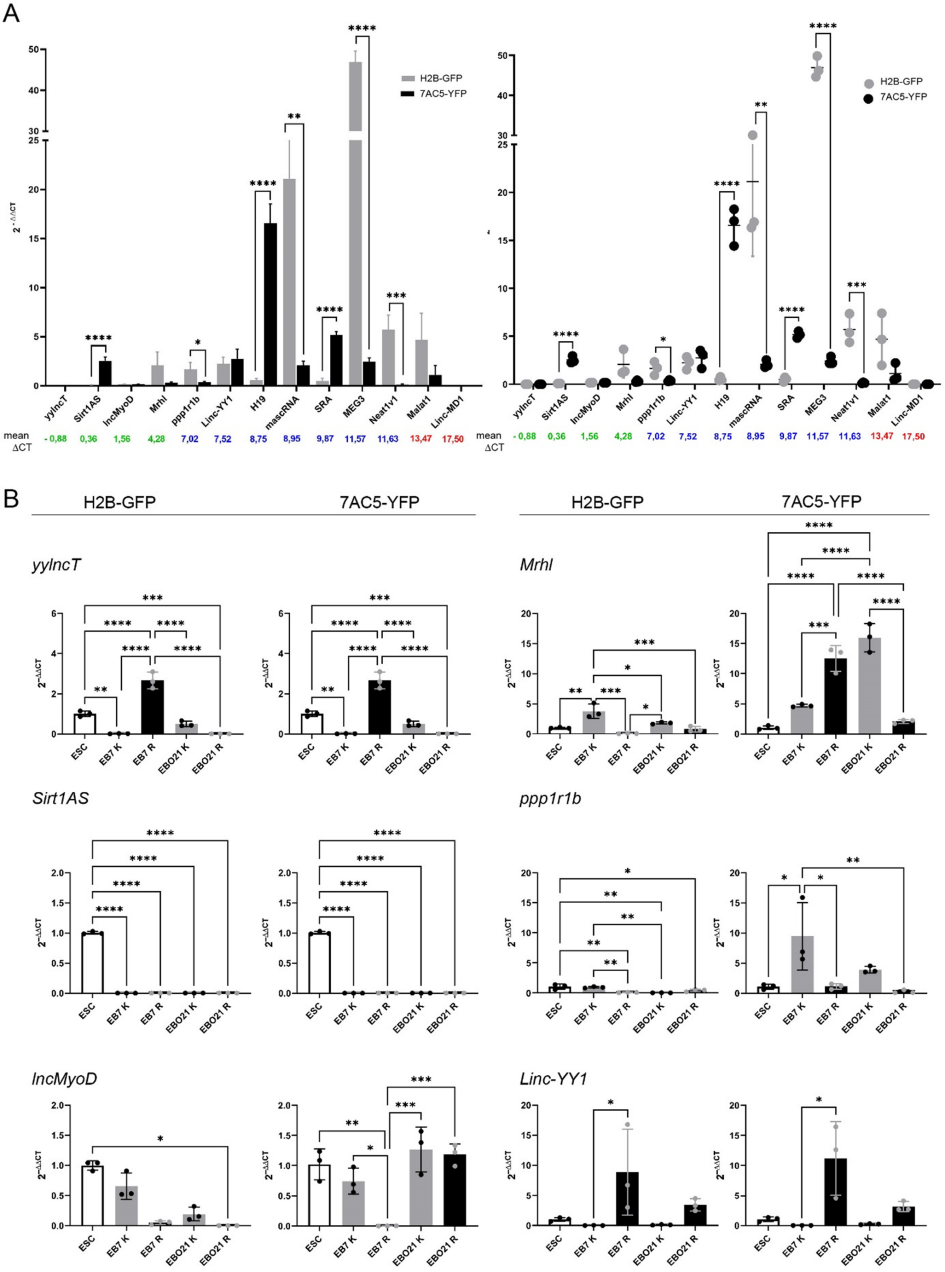
(**A**) Expression of selected lncRNAs in undifferentiated H2B-GFP and 7AC5-YFP ESCs; (**B**) Expression of selected lncRNAs in H2B-GFP and 7AC5-YFP cells analyzed at indicated time points.(**A**) The numbers in color indicate the mean ΔCT for the mouse embryo. The mean with standard deviation for ESCs is shown on the left graph while values obtained for each biological replicate are shown on the right graph. The stars show significant differences between H2B-GFP and 7AC5-YFP ESC cells in the level of expression of the same lncRNA; (**B**) K refers to cells cultured in control medium (grey columns). R refers to cells cultured in RA/ITS medium (black columns). Empty columns refer to undifferentiated ESCs. Mean with standard deviation as well as values obtained for each biological replicate are shown. Stars show significant differences between the indicated cells. *p<0.05,**p<0.01, ***p<0.001, ****p<0.0001. Each experiment was performed in 3 replicates.

**Fig. 3:**
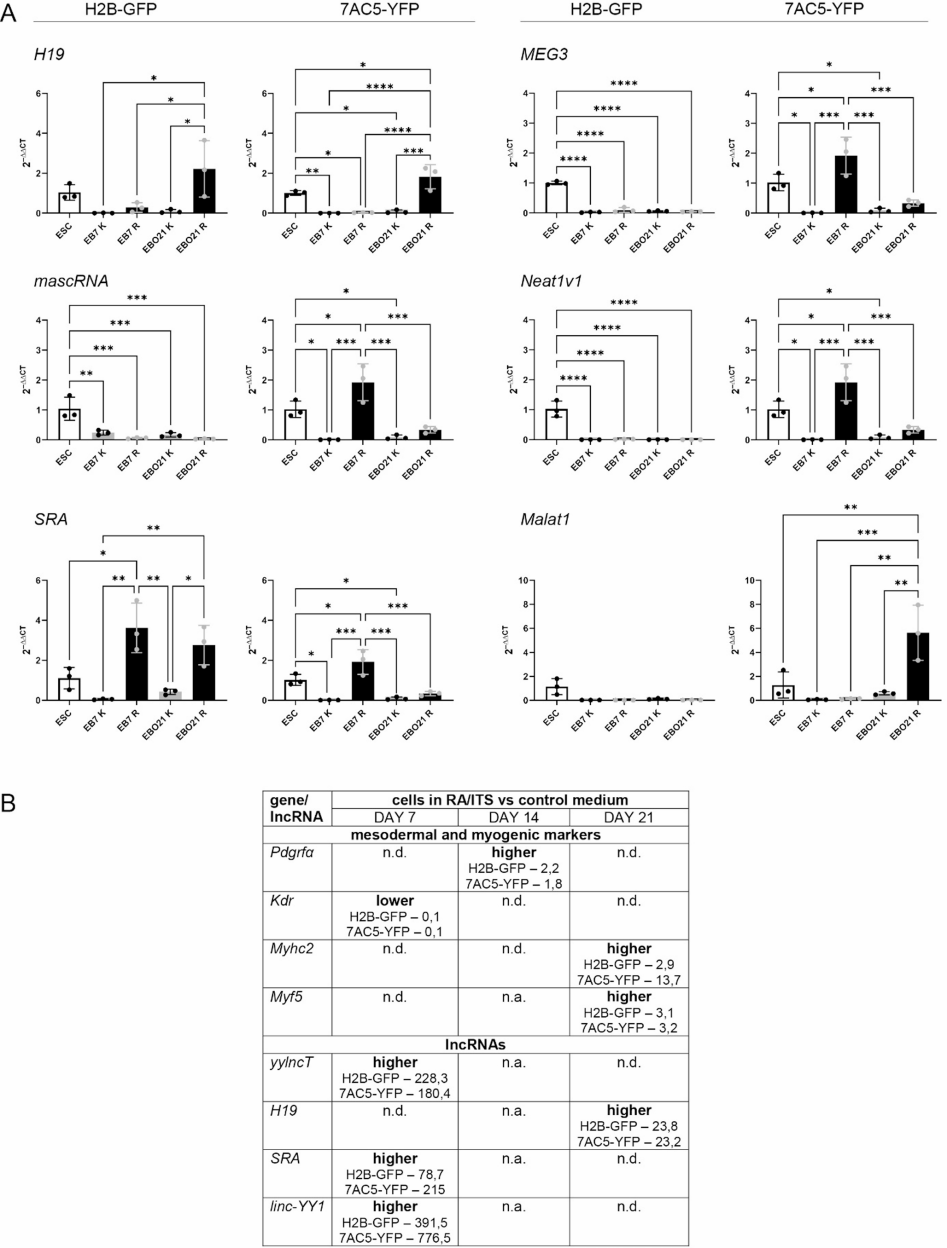
(**A**) Expression of selected lncRNAs in H2B-GFP and 7AC5-YFP cells analyzed at the indicated time points. (**B**). Summary of mesodermal and myogenic markers and lncRNAs expressed in the same pattern in differentiating (RA/ITS treated) ESCs compared to control ones. (**A**) K refers to cells cultured in control medium (grey columns). R refers to cells cultured in RA/ITS medium (black columns). Empty columns refer to undifferentiated ESCs. The mean with standard deviation as well as values obtained for each biological replicate are shown. Stars refer to all significant differences found between indicated, analyzed samples. *p<0.05, **p<0.01, ***p<0.001,****p<0.0001. Each experiment was performed in 3 replicates. (**B**) Only those changes that were significant in both ESC lines, *i.e.*, higher or lower in RA/ITS treated cells compared to cells cultured in control medium, are presented. Presented values refer to fold chance, i.e. mean RQ for cells cultured in RA/ITS medium divided by the mean RQ for cells cultured in control medium. n.d. - no significant differences, n.a. - not assessed.

Next, we compared the expression of lncRNAs in undifferentiated H2B-GFP and 7AC5-YFP ESCs (Fig. 2A). Calculated ΔCT from independent experiments, ΔCT mean values and standard error of the mean are presented in Tables S2 and S3. We also followed lncRNA level in differentiating ESCs, i.e., EBs on day 7, and EBOs at day 21 cultured in control or in RA/ITS medium (Figs. 2B and 3A). Calculated ΔCT from independent experiments, ΔCT mean values and standard error of the mean for such cells are presented in Tables S4-S7. Such analyzes were not previously performed. We found significant differences in the initial levels of analyzed lncRNAs between the ESC lines. The undifferentiated 7AC5-YFP ESCs presented higher levels of *Sirt1AS*, *H19*, and *SRA*, while the undifferentiated H2B-GFP ESCs were characterized by higher levels of *mascRNA*, *Neat1v1*, *ppp1r1b*, and *MEG3* (Fig. 2A). In regards to differentiating ESCs, we found a significantly increased level of *yylncT*, essential for mesoderm differentiation of cells in RA/ITS treated EB7 (both lines, Fig. 2B, Table S4-7). These cells were also characterized by significantly higher expression of the *Linc-YY1* and *SRA* - inducers specific for myogenic differentiation (Figs. 2B and 3A). H2B-GFP cells treated with RA/ITS were characterized by a higher level of *SRA* also on day 21 (EBO21; Fig. 3A). The expression of other myogenic differentiation-promoting lncRNA, i.e., *H19*, was higher on day 21 in both cell lines treated with RA/ITS (Fig. 3A). Additionally, elevated expression of *Mrhl*,* mascRNA*,* MEG3*, and *Neat1v1* was found in 7AC5-YFP cells analyzed on day 7 of RA/ITS culture, while *Malat1* – at day 21 in such cells (Figs. 2B and 3A). On the other hand, control 7AC5-YFP EB7 were characterized by the elevated expression of *lncMyoD*, which was described as promoting myogenic differentiation (Fig. 2B). In both cell lines cultured in control medium, *ppp1r1b* expression was higher on day 7 (Fig. 2B). Expression of *Mrhl*, which is engaged at early stages of ESC differentiation, was also significantly higher in control cells; however, in the case of H2B-GFP it was stage EB7, while in 7AC5-YFP - EBO21 (Fig. 2B). *Sirt1AS* expression was not detected in differentiating ESCs (Fig. 2B). The expression of *Linc-MD1* was not detected in any samples. All indicated information were gathered and summarized in Table 1 showing comparison of lncRNA expression in H2B-GFP and 7AC5-YFP ESCs on day 7 or 21 of culture either in control or RA/ITS medium.

**Table 1:** Comparison of LncRNA expression in H2B-GFP and 7AC5-YFP ESCs on day 7 or 21 of culture either in control or RA/ITS medium. Values refer to fold change (FC) calculated by dividing the mean RQ (2^−ΔΔCT^) obtained for the sample with higher gene expression by the mean RQ for the sample with lower gene expression. FC = fold change, n.d. = no significant difference

lncRNA	D7				D21			
	H2B-GFP		7AC5-YFP		H2B-GFP		7AC5-YFP	
	control	RA/ITS	control	RA/ITS	control	RA/ITS	control	RA/ITS
*yyylncT*		higher FC: 228,3 *p* < 0,0001		higher FC: 180,4 *p* < 0,0001	n.d.		n.d.	
*Sirt1AS*	n.d.		n.d.		n.d.		n.d.	
*lncMyoD*	n.d.		higher FC:347,6 *p* = 0,0216		n.d.		n.d.	
*Mrhl*	higher FC: 55,2 *p* = 0,001			higher FC: 2,6 *p* = 0,004	n.d.		higher FC: 7,7 *p* < 0,0001	
*ppp1r1t*	higher FC: 0,9 *p* = 0,0053		higher FC: 8,3 *P* = 0,0159		n.d.		n.d.	
*Linc-YY1*		higher FC: 391,5 *p* = 0,0191		higher FC: 776,5 *p* = 0,0102	n.d.		n.d.	
*H19*	n.d.		n.d.			higher FC: 23,8 *p* = 0,0189		higher FC: 23,2 *p* = 0,0001
*mascRNA*	n.d.			higher FC: 215 *p* = 0,0001	n.d.		n.d.	
*SRA*		higher FC: 78,7 *p* = 0,0013		higher FC: 215 *p* = 0,0001		higher FC: 6,5 *p* = 0,0227	n.d.	
*MEG3*	n.d.			higher FC: 215 *p* = 0,0001	n.d.		n.d.	
*Neat1v1*	n.d.			higher FC: 215 *p* = 0,0001	n.d.		n.d.	
*Malat1*	higher FC: 5,3 *p* = 0,0361		n.d.		n.d.			higher FC: 9,6 *p* = 0,0021

Next, we looked at the impact of culture conditions on lncRNA induction over time. For both lines, an increase in the level of *yylncT* and *SRA* was observed between ESCs and EB7, while *H19* was elevated between the EB7 and EBO21 stages in RA/ITS-treated cells (Figs. 2B and 3A). Additionally, an increase in the level of other lncRNAs was observed in 7AC5-YFP cells between days 0 and 7 (*Mrhl*,* mascRNA*,* MEG3* and *Neat1v1*) as well as between days 7 and 21 (*lncMyoD* and *Malat1*; Figs. 2B and 3A). Control culture conditions induced the expression of *ppp1r1b* in 7AC5-YFP EB7 and *Mrhl* in H2B-GFP EB7 compared to ESCs (Fig. 2B). Furthermore, *Mrhl* expression increased between day 7 and 21 in cells from the 7AC5-YFP line (Fig. 2B). All indicated information were gathered and summarized in Table 2 showing changes in lncRNA expression in H2B-GFP and 7AC5-YFP ESCs on day 7 or 21 of the culture either in control or RA/ITS medium, compared to undifferentiated ESCs or cells at earlier stages of differentiation (day 7 versus 21), cultured under the same conditions.

**Table 2:** Changes in LncRNA expression in H2B-GFP and 7AC5-YFP on day 7 or 21 of culture either in control or RA/ITS medium, compared to undifferentiated ESCs or cells at earlier stages of differentiation (day 7 versus 21), cultured under the same conditions. Fold change (FC) was calculated by dividing the mean RQ obtained for the sample with higher gene expression by the mean RQ for the sample with lower gene expression. FC = fold change, n.d. = no significant difference

lncRNA	H2B-GFP ESC				7AC5-YFP ESC			
	control		RA/ITS		control		RA/ITS	
	D7 to ESCs	D21 to D7	D7 to ESCs	D21 to D7	D7 to ESCs	D21 to D7	D7 to ESCs	D21 to D7
*yyylncT*	n.d.	n.d.	higher FC: 3,8 *p* = 0,0002	lower FC:1562,1 *p* < 0,0001	lower FC: 68,1 *p* = 0,001	n.d.	higher FC: 2,6 *p* < 0,0001	lower FC:9768,6 *p* < 0,0001
*Sirt1AS*	lower FC: 3578,6 *p* < 0,0001	n.d.	lower FC: 3853,8 *p* < 0,0001	n.d.	lower FC:8213,1 *p* < 0,0001	n.d.	lower FC: 19269,23 *p* < 0,0001	n.d.
*lncMyoD*	n.d.	n.d.	n.d.	n.d.	n.d.	n.d.	lower FC:478,3 *p* = 0,0026	higher FC: 553,9 *p* = 0,0008
*Mrhl*	higher FC: 3,8 *p* = 0,0011	lower FC: 2,1 *p* = 0,0123	n.d.	n.d.	n.d.	higher FC: 3,4 *p* < 0,0001	higher FC; 12,1 *p* < 0,0001	lower FC: 6,1 *p* < 0,0001
*ppp1r1t*	n.d.	lower FC: 21,8 *p* = 0,0032	lower FC: 9,9 *p* = 0,0023	n.d.	higher FC: 8,8 *p* = 0,015	n.d.	n.d	n.d.
*Linc-YY1*	n.d.	n.d.	n.d	n.d.	n.d.	n.d.	n.d.	n.d.
*H19*	n.d.	n.d.	n.d.	higher FC: 7,8 *p* = 0,0327	lower FC:878,7 *p* = 0,0086	n.d.	lower FC: 32,8 *p* = 0,0104	higher FC: 59,4 *p* < 0,0001
*mascRNA*	lower FC: 4,3 *p* = 0,0019	n.d.	lower FC: 16,7 *p* = 0,0004	n.d.	lower FC:114,5 *p* = 0,016	n.d.	higher FC: 1,9 *p* = 0,0326	lower FC: 5,7 *p* = 0,0007
*SRA*	n.d.	n.d.	higher FC: 3,3 *p* = 0,0148	n.d.	lower FC:114,5 *p* = 0,016	n.d,	higher FC: 1,9 *p* = 0,0326	lower FC: 5,7 *p* = 0,0007
*MEG3*	lower FC: 42,4 *p* < 0,0001	n.d.	lower FC: 10,2 *p* < 0,0001	n.d.	lower FC:114,5 *p* = 0,016	n.d.	higher FC: 1,9 *p* = 0,0326	lower FC: 5,7 *p* = 0,0007
*Neat1v1*	lower FC:301,8 *p* < 0,0001	n.d.	lower FC: 69,3 *p* < 0,0001	n.d.	lower FC:114,5 *p* = 0,016	n.d.	higher FC: 1,9 *p* = 0,0326	lower FC: 5,7 *p* = 0,0007
*Malat1*	n.d.	n.d.	n.d.	n.d.	n.d.	n.d.	n.d.	higher FC: 49,8 *p* = 0,0011

Since RA/ITS supplemented medium decreased the level of *Kdr* (a marker of mesoderm differentiating into cardiomyocytes) and at the same time increased the expression of *Pdgfra* (a marker of mesoderm differentiating into myogenic precursors), *Myhc2* (characteristic of maturing myofibers), and *Myf5* (one of MRFs, i.e., muscle regulatory factors, characteristic for skeletal muscle lineage), as well as such lncRNAs as *yylncT*, *Linc-YY1*, *SRA*, and *H19* in both cell lines as summarized in Fig. 3B, we decided to perform subsequent experiments using such culture conditions. As described above, the indicated differences at mRNA and lncRNA level were accompanied by significantly more cells positive for skMYHC and negative for cardiac troponin T in EBOs generated in RA/ITS supplemented medium.

### Impact of *miR145* or *miR181* on ESC myogenic differentiation and lncRNA expression

**Fig. 4:**
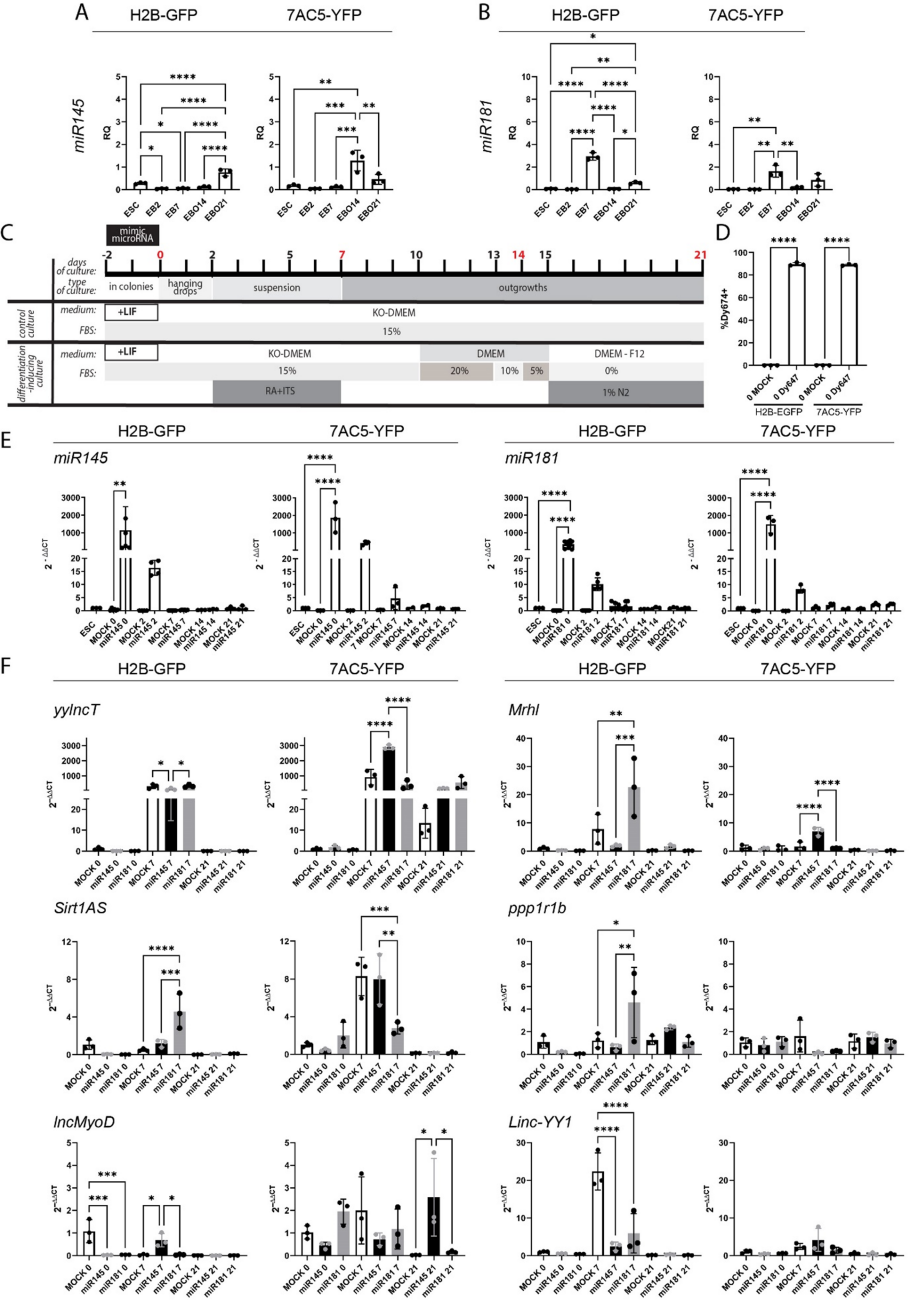
(**A**,**B**) Endogenous expression of * miR145* and *miR181* in H2B-GFP and 7AC5-YFP cells cultured in RA/ITS supplemented medium and analyzed at the indicated time points. (**C**) Schematic diagram showing the protocol used to induce the differentiation of ESCs; (**D**) Proportion of ESCs positive for miRNA Mimics (Dy647) labeled with FITC established by flow cytometry; (**E**) Expression of * miR145* and *miR181* in control non-treated ESCs, MOCK treated, and *miR145* or *miR181* transfected H2B-GFP and 7AC5-YFP ESCs analyzed at indicated time-points; (**F**) Expression of selected lncRNAs in H2B-GFP and 7AC5-YFP ESCs after treatment with either *miR145* or *miR181*, or in MOCK cells cultured in RA/ITS medium and analyzed at the indicated time points. Empty columns refer to MOCK cells, black columns to cells treated with *miR145*, grey columns to cells treated with *miR181.* The mean with standard deviation as well as values obtained for each biological replicate are shown. The stars show significant differences between cells analyzed at the same time point (MOCK cells vs cells treated with *miR145*, MOCK cells vs cells treated with *miR181*, cells treated with *miR145 *vs cells treated with *miR181*).*p<0.05, **p<0.01, ***p<0.001, ****p<0.0001. Each experiment was performed in 3 replicates

To assess whether the miRNAs used by us in the previous studies, i.e.,* miR145* or *miR181*, enhance ESC myogenic differentiation by interplaying with lncRNAs, we first examined the endogenous level of these miRNAs in H2B-GFP and 7AC5-YFP differentiating in RA/ITS-supplemented medium (Fig. 4A, B). Then we transiently overexpressed these miRNAs in ESCs ([9, 47], as shown on experimental scheme in Fig. 4C. Endogenous presence of both miRNAs was confirmed in analyzed cells, with the highest level of *miR145* in H2B-GFP EBO21 and 7AC5-YFP EBO14 (Fig. 4A). The highest level of endogenous *miR181* was found in EB7 in both cell lines (Fig. 4B). After miRNA overexpression we first assessed transfection efficiency, using fluorescently labeled control RNA - Dy647. We analyzed cells treated with transfection reagent only (MOCK) and Dy647 transfected cells 48 h post transfection. The proportion of fluorescently labelled cells reached 89.75% for H2B-GFP ESCs and 90.68% for 7AC5-YFP (Fig. 4D). Next, we analyzed the levels of *miR145* and *miR181* in non-transfected ESCs (control cells) and cells analyzed 48 h after transfection, i.e., at day 0 of the experiment as well as 2, 7, 14 and 21 days later (experimental scheme in Fig. 4C). Regardless of cell line and miRNA type, the significantly high level of both analyzed miRNAs was found in transfected cells at day 0 (Fig. 4E). To verify the impact of such treatment on the progression of myogenic differentiation, we estimated the level of previously analyzed and useful markers, such as *Pdgfra*, *Myhc2*, *Myhc3*, and *Myf5.* This allowed us to reveal that cells from both lines, treated with *miRNAs*, were characterized with significantly higher level of *Pdgfra* and *Myhc2*, as well as lower level of *Myhc3*, embryonic MyHC, characteristic for less advanced stages of myogenesis (Fig. S4A). Expression of *Myf5* was cell line specific, i.e., it was significantly higher in miRNA overexpressed H2B-GFP cells, regardless of miRNA type, in comparison to MOCK cells while in 7AC5-YFP it was opposite (Fig. S4A). Obtained results indicate that miRNA overexpression indeed induced mesodermal and myogenic differentiation of treated ESCs but to different range and pace as suggested by found differences. To verify the functional outcome of myogenic differentiation of ESCs induced by miRNA overexpression, we co-cultured transfected ESCs with differentiating C2C12 myoblasts and assessed formation of myotubes, as well as the fusion index. Myotubes were generated in all types of the co-culture, i.e., C2C12 with ESCs transfected with either *miR145* or *miR181*, as well as in control variant with MOCK ESCs (Fig. S4B). Assessment of fusion index values revealed that it was significantly elevated in co-cultures of C2C12 myoblasts with transfected ESCs, regardless of miRNA type used and ESC line analyzed, in comparison to control co-cultures with MOCK ESCs (Fig. S4C). This indicates that miRNA overexpression indeed promoted myogenic differentiation of ESCs, including enhancement of their ability to fuse with myoblasts.

**Fig. 5:**
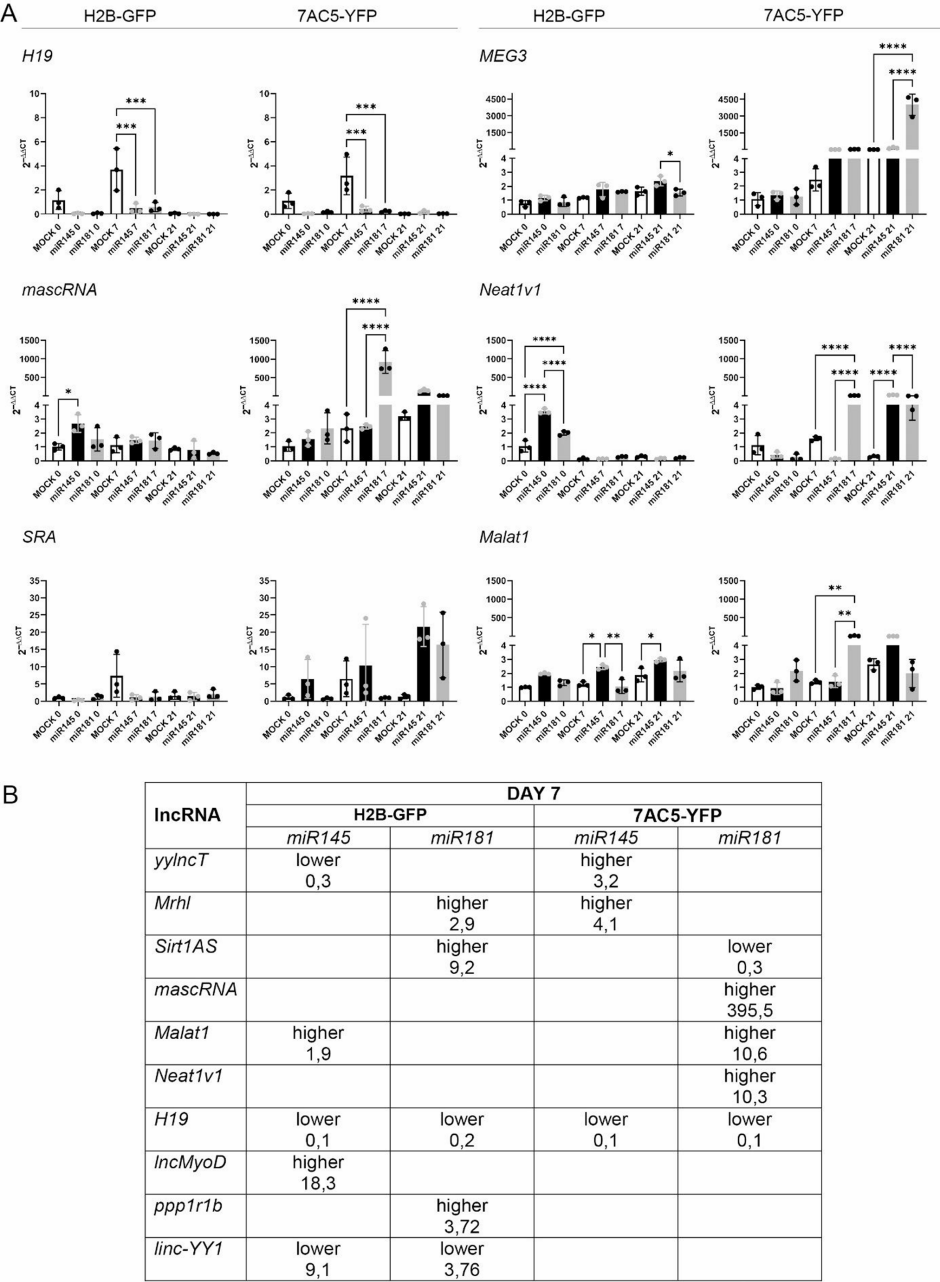
(**A**) Expression of selected lncRNAs in H2B-GFP and 7AC5-YFP ESCs after treatment with either *miR145* or *miR181*, or in MOCK cells, cultured in RA/ITS medium and analyzed at the indicated time points. Empty columns refer to MOCK cells, black columns to cells treated with *miR145*, grey columns to cells treated with *miR181.* The mean with standard deviation as well as values obtained for each biological replicate are shown. The stars show significant differences between cells analyzed at the same time point (MOCK cells vs cells treated with *miR145*, MOCK cells vs cells treated with *miR181*, cells treated with *miR145 *vs cells treated with *miR181*).*p<0.05, **p<0.01, ***p<0.001, ****p<0.0001. Each experiment was performed in 3 replicates; (**B**) Changes in lncRNA expression in H2B-GFP and 7AC5-YFP on day 7 after induction of *miR145* or *miR181* overexpression procedure, compared to MOCK cells, cultured under the same conditions (*i.e., *RA/ITS treatment). Presented values refer to fold chance, i.e. mean RQ for cells cultured in RA/ITS medium and treated with indicated miRNA, divided by the mean RQ for MOCK cells cultured in RA/ITS medium

Finally, using ENCORI Encyclopedia of RNA Interactomes we were able to find several predicted miRNA-lncRNA interactions: *miR181a-5p* contains sequence compatible with sequences present in *Malat1*, *MEG3*, and *Neat1v1* while *miR145a-5p* contains sequence compatible with *Neat1v1*. Based on that we presumed that the levels of at least these lncRNAs might be affected by the overexpression of miRNA studied by us. To this end, we compared the levels of these and previously selected by us other lncRNAs in ESCs treated with transfection reagent (MOCK) or transfected for 48 h with miRNAs (either *miR145* or *miR181*). ESCs were analyzed immediately after miRNA transfection, that is, on day 0, and also on day 7 (EBs), and day 21 of differentiation (EBOs; Figs. 4F and 5A). Calculated ΔCT from independent experiments, ΔCT mean values and standard error of the mean are presented in Table S8-S11. Both cell lines differed in the lncRNA initial levels (Fig. 2A). lncRNA levels were also different in the MOCK ESCs analyzed on day 0 (Table S8-11). However, some common features were distinguishable.

Regarding the mesoderm controlling lncRNAs, the expression of *yylncT* was highest on day 7 of differentiation, i.e., on EB stage. On day 7, *miR145* downregulated the levels of *yylncT*, compared to the control (MOCK) in H2B-GFP cells, while in 7AC5-YFP significantly elevated this RNA (Fig. 4F). At this time point, the level of *yylncT* was similar in MOCK and *miR181* treated cells of both lines. *Mrhl* expression was low on day 0 in all experimental groups, regardless of the cell line. On day 7 *miR181* significantly up-regulated this lncRNA but only in H2B-GFP, while in the case of 7AC5-YFP cells it was induced by *miR145* (Fig. 4F). LncRNA involved in MPC specification and myoblast proliferation, that is, *Sirt1AS*, was also up-regulated by *miR181* in H2B-GFP but down-regulated in 7AC5-YFP cells, on day 7 (Fig. 4F). Differences between both ESC lines and used miRNAs were also observed for other lncRNAs that control proliferation and early stages of myoblast differentiation, that is, *mascRNA* and *Malat1* (Fig. 5A). In this case, up-regulation of both indicated lncRNAs was found in both ESC lines, however, in the case of H2B-GFP it was induced by *miR145*, while in the case of 7AC5-YFP it was induced by *miR181* (Fig. 5A). Furthermore, in the case of *Neat1v1*, upregulation of this lncRNA was found in H2B-GFP cells treated with *miR145* (day 0), while in 7AC5-YFP cells after treatment with *miR181* (day 7) and additionally by *miR145* on day 21 (Fig. 5A).

Next, lncRNAs specific for differentiating myocytes - *H19*,* lncMyoD*, and *SRA* - were studied. Treatment with *miR145* or *miR181* resulted in the drop in *H19* expression in both cell lines on day 7 (Fig. 5A). *SRA* expression was not affected by *miR145* or *miR181* (Fig. 5A). On day 0, the expression of *lncMyoD* was lower in H2B-GFP treated with *miR145* or *miR181*, compared to MOCK, however, on day 7 *miR145* increased its level (Fig. 4F). *MiR145* also upregulated *lncMyoD* in 7AC5-YFP cells, but on day 21 (Fig. 4F). Finally, we focused on *ppp1r1b*,* Linc-YY1*, and *MEG3*, which are characteristic for the later stages of myogenic differentiation. The expression of *ppp1r1b and Linc-YY1* was increased by *miR181* but only in H2B-GFP cells, on day 7 (Fig. 4F). In the case of *MEG3*, upregulation of this lncRNA was observed on day 21, but again, in H2B-GFP cells it was triggered by *miR145*, while in 7AC5-YFP cells – by *miR181* (Fig. 5A). Obtained results indicated that in case of transient up-regulation of *miR145* or *miR181*, changes in lncRNA expression were detectable primarily on day 7 of differentiation. We summarized these changes in Fig. 5B, however, they were diverse for different ESC lines and miRNA used except *H19* which decreased in case of both ESC lines and miRNA type used (Fig. 5B). Figures 4F and 5A document significant differences only between MOCK and miRNA transfected cells analyzed in the same time point. Complete statistical results showing all significant differences observed between the analyzed cells are presented on Figures S5 and S6. Altogether, our results for the first time document the differential pattern of selected lncRNAs during myogenic differentiation of two ESC lines overexpressed with 2 types of miRNAs, i.e.,* miR145* and *miR181*, suggesting stage and cell line specific interplay between these miRNAs and lncRNAs in differentiating ESCs.

## Discussion

Studies involving PSCs, such as ESCs or iPSCs, are always burdened by the fact that these cell lines may differ in many aspects. Multiple previous studies pinpointed the fact that even cell lines derived within the same institute could present very diverse characteristics (e.g [54, 55]). Thus, when studying PSCs, one cannot expect that the experimental settings used in other studies will result in the same outcome. In our previous study, in which we investigated mouse ESC myogenic differentiation, we used several independent cell lines. Some of them were commercially available, some of them were derived by us [9, 47]. Each of the in-house-derived ESC lines was characterized by us to show that they present differentiation potential and can be used in analyzes of myogenic differentiation [9, 47, 48, 57–59]. In the current study we used two other ESC lines, i.e., H2B-GFP and 7AC5-YFP. As mentioned before, such choice was made as experiments described in the current article were part of the bigger project encompassing assessment of the miRNA impact at ESC differentiation both in vitro and in vivo. Using cells expressing fluorescent proteins allowed us to trace their progeny during in vivo experiments involving cell transplantation into regenerating muscles (Archacka et al., submitted). The method for inducing ESC myogenic differentiation, selected for the experiments described in the current article, relied on our previous experiments [9, 47, 48]. However, as we mentioned previously, due to the variability in PSC phenotypes, we had to verify whether RA/ITS-containing medium in the same way induces H2B-GFP and 7AC5-YFP ESCs as in the case of B8 ESCs [9, 47]. RA/ITS driven differentiation elevated *Pdgfrα* expression and decreased *Kdr* expression in H2B-GFP and 7AC5-YFP ESCs used in this study. Furthermore, *Myhc2* and *Myf5* were upregulated. Thus, we have proven that both indicated cell lines react as previously described [9, 47] to the differentiation protocols we used.

The key aim of the current study was to reveal expression pattern of lncRNAs, known as mesodermal and myogenic regulators, during myogenic differentiation of ESCs induced by RA/ITS treatment as well as after use of additional stimulus, i.e., the overexpression of either *miR145* or *miR181*. Up-regulation of myogenic markers and enhanced myotube formation by custom-derived B8 mouse ESCs transfected with indicated miRNAs was shown by Bem et al. Increase in the ability to fuse in miRNA transfected ESC was also noticed by us, regardless of cell line or miRNA type used. Such effect was probably related to changes in cytoskeleton organization and fusion regulators such as versican/Vers, PGK1, ENO-1 or PI3K-AKT-mTOR which were revealed by microarray analysis in ESCs directly after overexpression of *miR181* and described in details in parallel study (Romanczuk et al., submitted). Moreover, in vivo experiments revealed that muscles injected with *miR181* overexpressing ESCs were characterized by elevated level of *Itga3* and *Cd9*, important regulators of myogenic cell migration, fusion, and differentiation [60], what probably contributed to enhanced engraftment of such cells observed in transplanted muscles (Archacka et al., submitted). Such effect could also, at least partially, result from enhanced expression of myogenic markers, such as *Pdgfra* and *Myhc2*, which was noticed by us in miRNA treated ESCs, regardless of cell line or miRNA type used.

Significant heterogeneity was found by us in lncRNA landscape: in undifferentiated ESCs, cells undergoing RA/ITS myogenic differentiation as well as those treated with miRNA. In the initial analysis we confirmed that all 13 lncRNAs, selected and analyzed by us as potential markers of PSC mesodermal/myogenic differentiation, were present in E13.5 mouse embryo and at least some of them in proliferating and differentiating C2C12 myoblasts, as well as in ESCs, both undifferentiated and differentiating in EBs and EBOs. At day 13.5 of mouse development, the formation of all organs and tissues is progressing, and for this reason, material isolated from such embryos serves as a good positive control for mRNA, protein, or ncRNA detection. Among the lncRNAs, which level was highest - as judged by ΔCT values - was *yylncT*, i.e., the one which regulates level of *T* expression in the primitive streak and the mesoderm. Among the abundant lncRNAs was also another regulator of early mesoderm differentiation - *Mrhl*, as well as *Sirt1AS* and *lncMyoD. Malat* and *Linc-MD1*, on the other hand, were among those with the lowest ΔCT values. *SirtAS*,* Mrhl*, and *lncMyoD* dominated also in proliferating C2C12 myoblasts, while *mascRNA* and *SRA* in differentiating ones. Thus, we confirmed the findings that *Sirt1AS* promotes myoblast proliferation [31]. *lncMyoD* was previously shown to be involved in myogenic differentiation [42, 43], however, we also detected it in proliferating C2C12 myoblasts. For the first time *Mrhl*, which is involved is the early germ layer specification [26], was documented by us also in proliferating myoblasts. *SRA* is known as lncRNA involved in MYOD activation and binding; therefore, its high level in differentiating myoblasts was expected and indeed found by us. On the other hand, *mascRNA* has been previously detected in proliferating rather than differentiating cells (e.g [61]), and no data on its potential involvement in myogenic differentiation was available so far. Its up-regulation, as well as *Malat1*, which is encoded in the same gene as *mascRNA*, was detected by us during C2C12 cell differentiation. *Malat1* was previously shown to be upregulated in differentiating mouse and human myoblasts [62] and its silencing in C2C12 cells prevented their fusion and myotube formation [63, 64]. However, other studies also suggested the involvement of *Malat1* in the regulation of myoblast [27, 65], as well as other cell types, e.g., cancer cells, proliferation (e.g [61]). Thus, it is possible that this particular RNA acts at different stages of myogenesis. Other lncRNAs, but one, i.e., *Linc-MD1*, were detectable in both proliferating and differentiating C2C12 cells.

Assessment of lncRNA expression in undifferentiated ESCs revealed that most of them were already detectable in such pluripotent stem cells. However, only the levels of *lncMyoD*, *Mrhl*, *Linc-YY1*, and *Malat1* were comparable between the two cell lines studied. In both ESC lines, *Sirt1AS* engaged in cell proliferation and inhibition of differentiation, was expressed at the highest level. H2B-GFP ESCs expressed significantly more *mascRNA*,* MEG3*, and *Neat1v1*, while 7AC5-YFP ESCs were characterized by higher expression of *SRA* and already mentioned *Sirt1AS*. Such a different status of the lncRNAs could result in different ESC potential to respond to culture conditions and miRNA overexpression. However, as already mentioned both cell lines presented common characteristics during RA/ITS induced myogenic differentiation, that is, they were characterized by elevated level of *Pdgfra*, *Myhc2*, and *Myf5*, as well as were able to form myotubes and myofibers when differentiating into EBOs, as described in the current article. This was in line with previous results by us, e.g [9, 47, 58, 66])., and others showing that despite the observed variability at the molecular level, PSCs manifest a characteristic allowing reliable conclusions on the mechanisms governing their pluripotency and differentiation. It also underlines, however, that to draw conclusions, more than one PSC line has to be analyzed using the same experiment settings.

Indicated culture conditions, i.e., RA/ITS treatment, also induced lncRNA changes common for both used ESC lines: it increased the mesoderm-associated *yylncT*, as well as myogenic differentiation-related *SRA*, *Linc-YY1*, and *H19* expression. However, changes at the lncRNA level were more diverse between analyzed ESC lines, since 7 of 12 analyzed lncRNAs elevated in 7AC5-YFP ESCs after myogenic differentiation induction, while H2B-GFP ESCs upregulated only 3 of them, compared to cells cultured under control conditions. Diverse changes in lncRNA expression in both analyzed ESC lines were also found after their transfection with *miR145* or *miR181*. It seems that each of the miRNAs tested differently affected the expression of the lncRNAs studied by us. In the response to *miR145* the level of *mascRNA*, *Neat1v1*, *Malat1*, and *lncMyoD* was elevated while *yylncT* and *Linc-YY1* decreased in H2B-GFP cells. In 7AC5-YFP *miR145* treatment also resulted in *Neat1v1* and *lncMyoD* elevation as well as increase in *yylncT* and *Mrhl* level. In case of *miR181* overexpression upregulation in *Mrhl*, *Sirt1AS*, and *ppp1rb* level was noticed in H2B-GFP cells while *Linc-YY1* was downregulated in such cells. In case of 7AC5-YFP *miR181* induced expression of *mascRNA*, *Malat1*, *Neat1v1*, and *MEG3* and down-regulated level of *Mrhl*, and *Sirt1AS*. *H19* was the only lncRNA studied by us which expression was downregulated by *miR145* and *miR181* in both ESC lines analyzed. This effect was unexpected as miRNA treatment enhanced myogenic differentiation of ESCs, as summarized above, and *H19* was previously described as lncRNA which expression is initiated by MYOD and elevated during differentiation of myogenic cells [36, 67]. Moreover, down-regulation of *H19* in porcine satellite cells restricts the expression of *Myod*, *Myog*, and *Myhc* [68]. Drop in *H19* expression in miRNA-induced ESCs could result from different functioning of *H19*-MYOD axis in such cells in comparison to differentiating myoblasts. This is supported by the study showing that overexpression of *H19* in P9 cells and elevation of this lncRNA in developing embryos of obese mice inhibits myogenesis by decreasing *Myf5*, *Myod1*, *Myog*, *Mef2c* and *Myhc3* expression [69]. Thus, it is possible that decreasing *H19* could in fact facilitate ESC myogenic differentiation. Such scenario is also further supported by study showing that *H19* operates as a molecular scaffold that facilitates effective association of K homology-type splicing regulatory protein (KSPR) with myogenin and other labile transcripts [70]. Dissociation of *H19* from KSRP abolishes KSRP-mediated myogenin degradation in undifferentiated C2C12 cells, and promotes this way their differentiation.

Information on *miR145*-*H19* or *miR181*-*H19* interactions potentially leading to observed *H19* drop are limited. In case of *miR145* such effect could be explained by the fact that this miRNA downregulates two pluripotency factors, OCT4 and SOX2, which were shown to influence *H19* level [71]. Next, *miR-145* has been shown to target DNMT3A, which can influence gene expression, what could potentially modulate DNA methylation at the *H19/IGF2* locus and affect its expression [72]. Bioinformatics analysis also found that *H19* can interact with *miR181*. Its overexpression led to the *miR181* downregulation [73] and knock-down increased *miR181* level [74], however, the reciprocal effect was not documented, so far. Similarly, potential interactions of used miRNAs with other lncRNAs analyzed in the current study, were not previously reported. As mentioned before, by using Encori (starBase or ENCORI: Decoding the Encyclopedia of RNA Interactomes) database we were able to find a few miRNA-lncRNA interactions: *miR181a-5p* has sequences compatible with *Malat1*, *MEG3*, *Neat1v1* while *miR145a-5p* with *Neat1v1*. In the current study we have observed increase in the level of *Neat1v1* in ESCs treated with each of the used miRNA but at different time-points for each ESC line. In case of *MEG3* its upregulation was observed only in 7AC5-YFP ESCs treated with *miR181* while in case of *Malat1* it was caused by *miR181* treatment in 7AC5-YFP but *miR145* in H2B-GFP cells. Interaction between *miR181a-5p* and *Neat1v1* was recently determined by dual-luciferase reporter gene assay in pancreatic cancer cells [75]. Predicted interaction between *miR181-5p* and *Malat1* was reported by Sun et al. [76]. However, this and other work indicated that it is *Malat1* to sponge *miR181*, not other way around [76, 77]. Thus, the fact that in our experiments overexpression of *miR181* resulted in the upregulation of *Malat1* in 7AC-YFP cells could be explained by compensatory mechanisms - more *miR181,* more *Malat1* needed to sponge it. Also *Neat* 1 was shown to regulate *miR181a* this way [78]. However, none of the above mentioned data came from the studies on pluripotent (ESCs, iPSCs) or even embryonic cells. Moreover, although available information suggest direct or indirect interactions between molecules studied by us, experimental data rarely document the lncRNAs are indeed downstream of miRNAs, but rather show opposite relation.

Observed differences in lncRNA pattern in undifferentiated ESCs as well as cells undergoing RA/ITS induced myogenic differentiation and most evidently during the response of two ESC lines to miRNA treatment, may result *inter alia* from diverse methylation level of analyzed lncRNA promoters as well as related factors. In general lncRNA promoters were shown to be more methylated than those of protein-coding genes, with implications for tissue differentiation [79]. However, detailed analyzes of the promoters of lncRNAs analyzed by us in different ESC lines are scarce or even absent. Abnormal hypermethylation within the *IGF2/H19* imprinting center was reported in seventeen primate ESC lines [80]. As a result of prolonged in vitro culture and passing, the human ESC line became biallelic with respect to *H19* without losing the gametic methylation imprint [81]. *MEG3* was listed among the genes which showed variable allelic expression between 22 human ESC lines, what of course could affect their phenotype [82]. But there is no data available on the differences between PSC lines regarding lncRNA promoter methylation, neither for *Neat1*, *lncMyod*, and others analyzed in our study. Such variability can undoubtedly affect PSC differentiation and was probably manifested also in our study.

## Conclusions

**Fig. 6:**
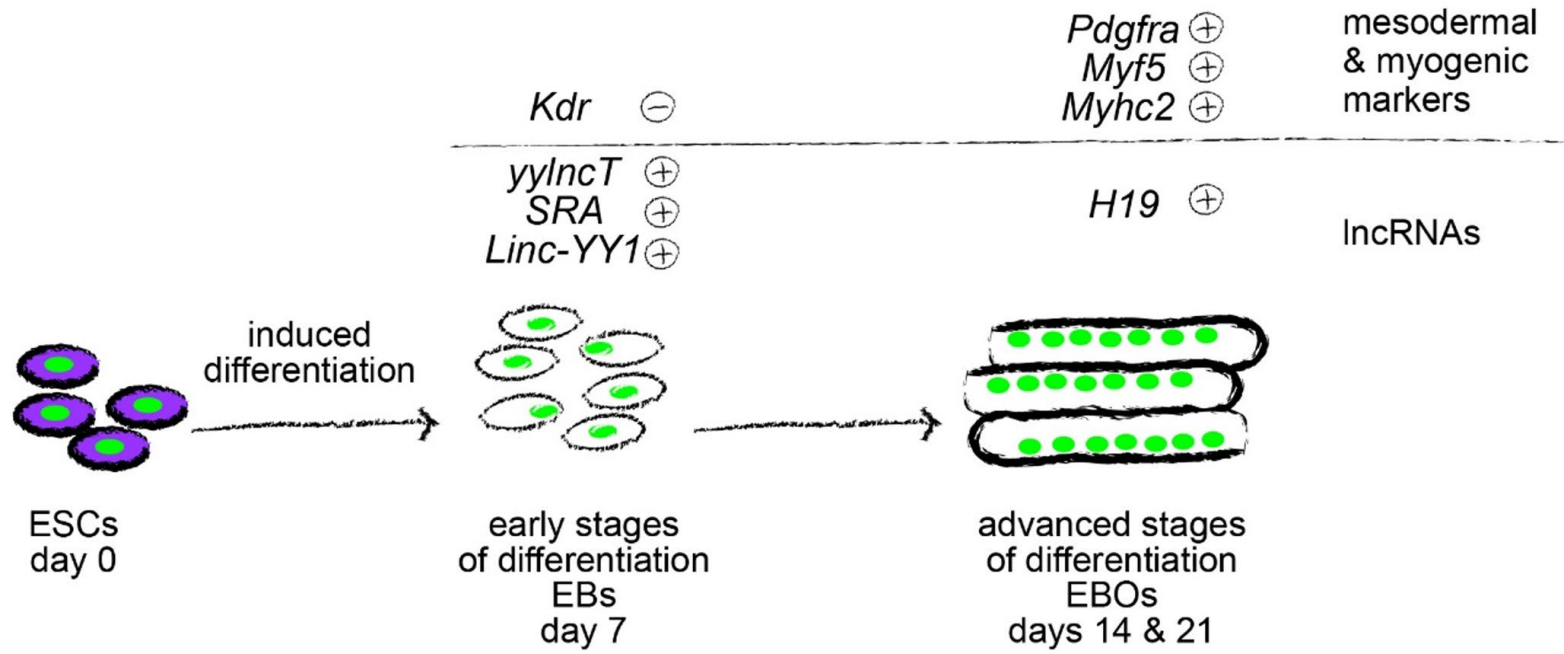
Graphical abstract. Summary of changes in analyzed mRNA and lncRNA expression in differentiating ESCs induced by RA/ITS treatment

RA/ITS mediated differentiation resulted in common changes in lncRNA expression detectable in both cell lines analyzed: up-regulation of *yylncT*,* H19*,* SRA*,* Linc-YY1* which is valuable as these lncRNAs can potentially serve as universal and early indicators of up-and-coming myogenic differentiation (Fig. 6). Both ESC lines studied by us also decreased *H19* in response to both *miR145* and *miR181*. However, the results of our study once again underscored the fact that ESC cell lines differ when exposed to various culture conditions and when exposed to treatments such as miRNA overexpression. Thus, during PSC differentiation studies each factor described as being a potential marker of certain cellular events should be treated with necessary caution. Further studies involving analyzes of multiple PSC lines are also necessary to verify potential markers indicated by us or expressed even in undifferentiated PSCs, which could help predict their ability to differentiate or confirm that it is indeed happening.

## Supplementary Information

Below is the link to the electronic supplementary material.

ESM 1 (DOCX 5.16 MB)

## Data Availability

Access to raw data is available on request.

## Declarations

### Cell Lines

No human cell lines were used in this study.
