# Supplementary material for: Mesoderm and myogenesis-related lncRNAs as Potential Markers of Myogenic Differentiation of Control and miR145 or miR181 Stimulated Mouse Pluripotent Stem Cells
Source: Stem Cell Rev Rep. 2025 Dec 17;22(2):889–909. doi: 10.1007/s12015-025-11034-z (PMC12858596; doi:10.1007/s12015-025-11034-z)
Supplement: Supplementary file 1 — (DOCX 5.16 MB) [file 12015_2025_11034_MOESM1_ESM.docx]

**SUPPLEMENTARY FIGURES**

**Figure S1. Expression of selected mesodermal markers in H2B-GFP and 7AC5-YFP ESC lines analyzed at the indicated time points.** K refers to cells cultured in control medium (grey columns). R refers to cells cultured in RA/ITS medium (black columns). Empty columns refer to undifferentiated ESCs, dashed columns – to EB2. Mean values and standard deviations as well as values obtained for each biological replicate are shown. Stars refer to all significant differences found between indicated, analyzed samples except cells cultured in different media and analyzed at the same time-point. *p<0.05, **p<0.01, ***p<0.001, ****p<0.0001. Each experiment was performed in 3 replicates.


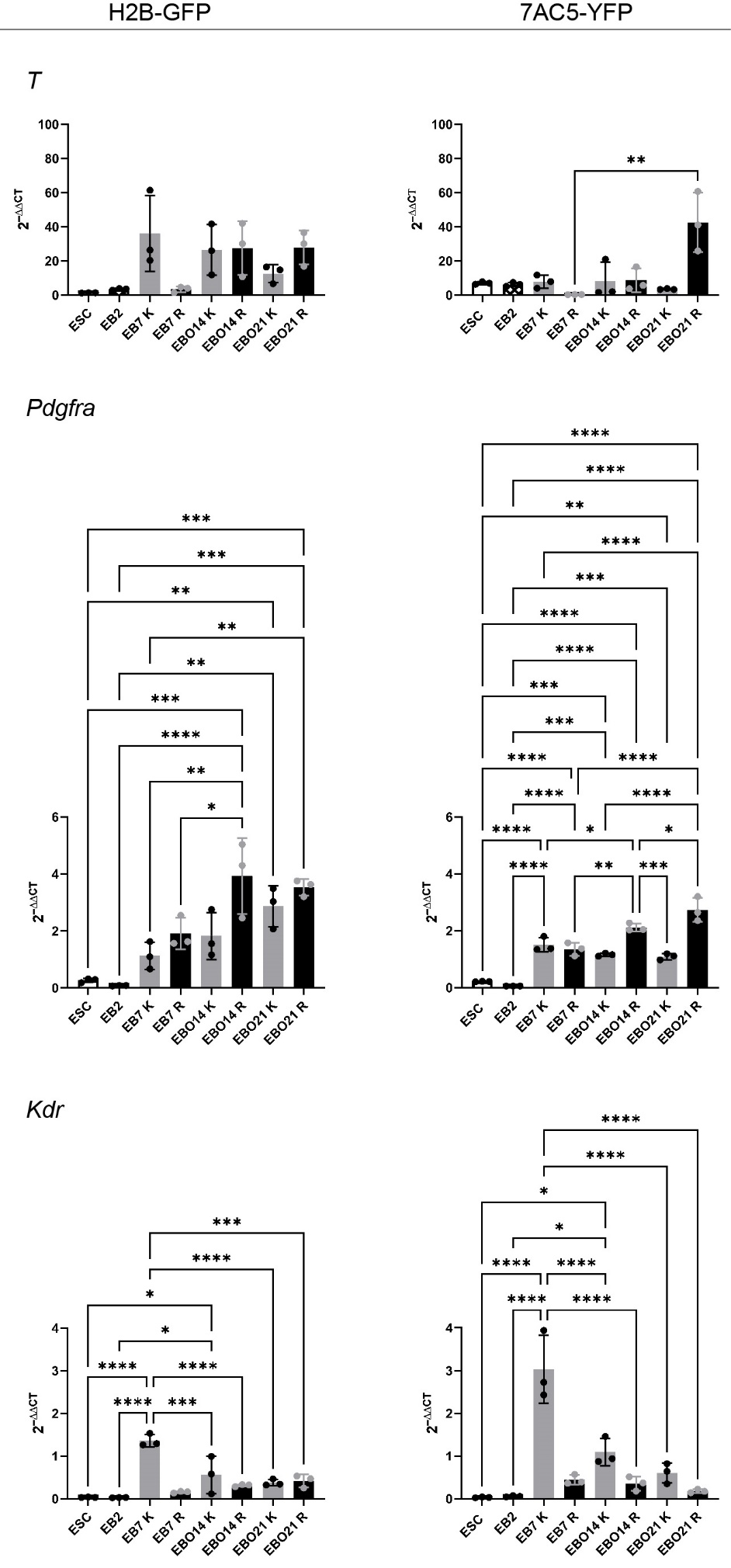


**Figure S2. Expression of selected myogenic markers in H2B-GFP and 7AC5-YFP ESC lines analyzed at the indicated time points.** K refers to cells cultured in control medium (grey columns). R refers to cells cultured in RA/ITS medium (black columns). Empty columns refer to undifferentiated ESCs, dashed columns – to EB2. Mean values and standard deviations as well as values obtained for each biological replicate are shown. Stars refer to all significant differences found between indicated, analyzed samples except cells cultured in different media and analyzed at the same time-point. *p<0.05, **p<0.01, ***p<0.001, ****p<0.0001. Each experiment was performed in 3 replicates.

**
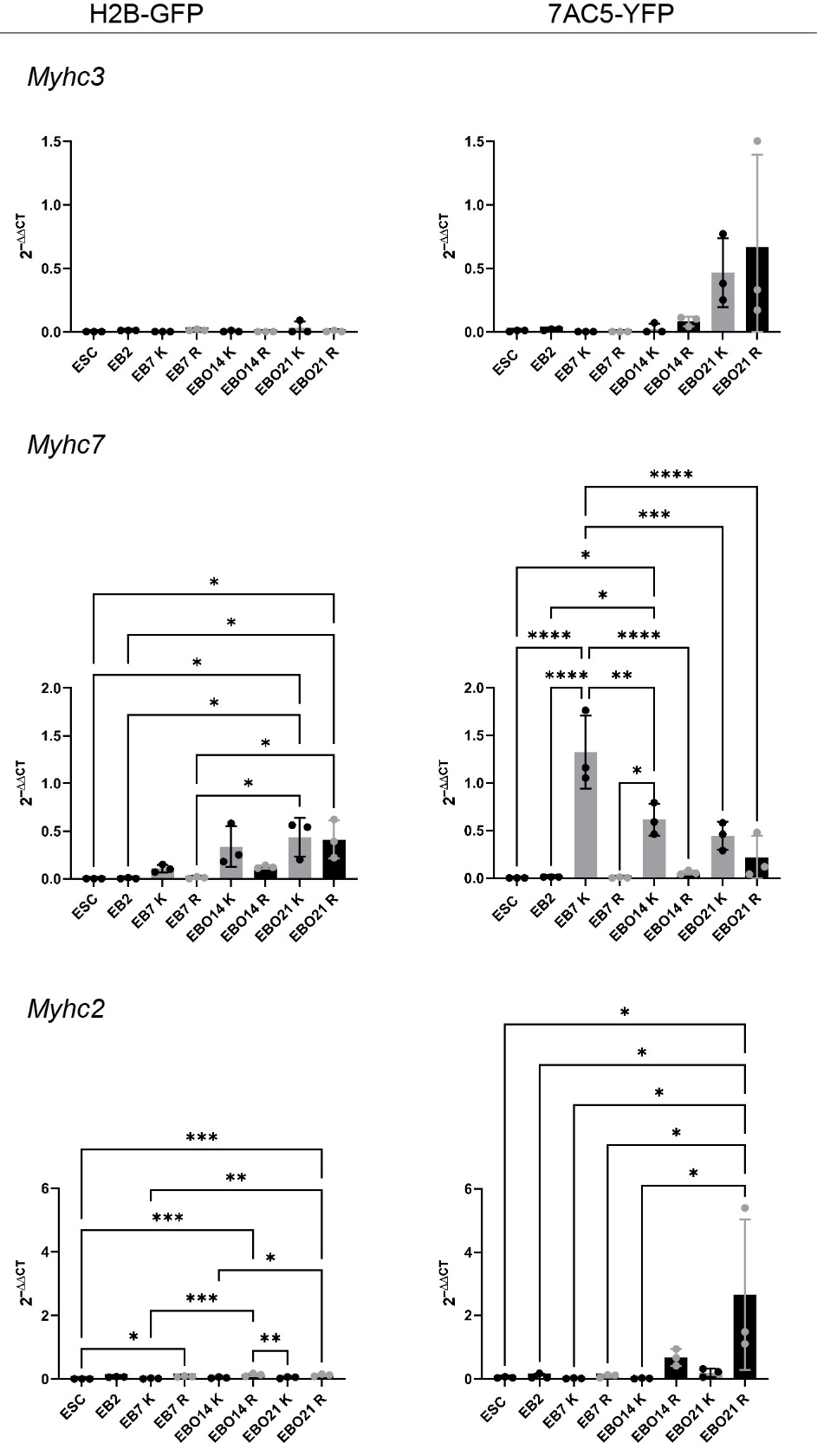
**

**Figure S3. Cell morphology and expression of *Myf5* in H2B-GFP and 7AC5-YFP EBOs generated in control and RA/ITS supplemented media.** (A) Microscopic images of EBOs generated from H2B-GFP and 7AC5-YFP EBOs cultured in control and RA/ITS supplemented media. Arrows indicate representative cells with myogenic morphology. Scale bar - 100 µm; (B) *Myf5* expression in cells cultured in control or RA/ITS medium and analyzed at indicated time-points. K refers to cells cultured in control medium (black columns). R refers to cells cultured in RA/ITS medium (empty columns). The mean values and standard deviation as well as values obtained for each biological replicate are shown. Stars refer to significant differences between cells cultured in different media (control vs RA/ITS) and analyzed at the same time-point. **p<0.01, ****p<0.0001. Each experiment was performed in 3-6 replicates.


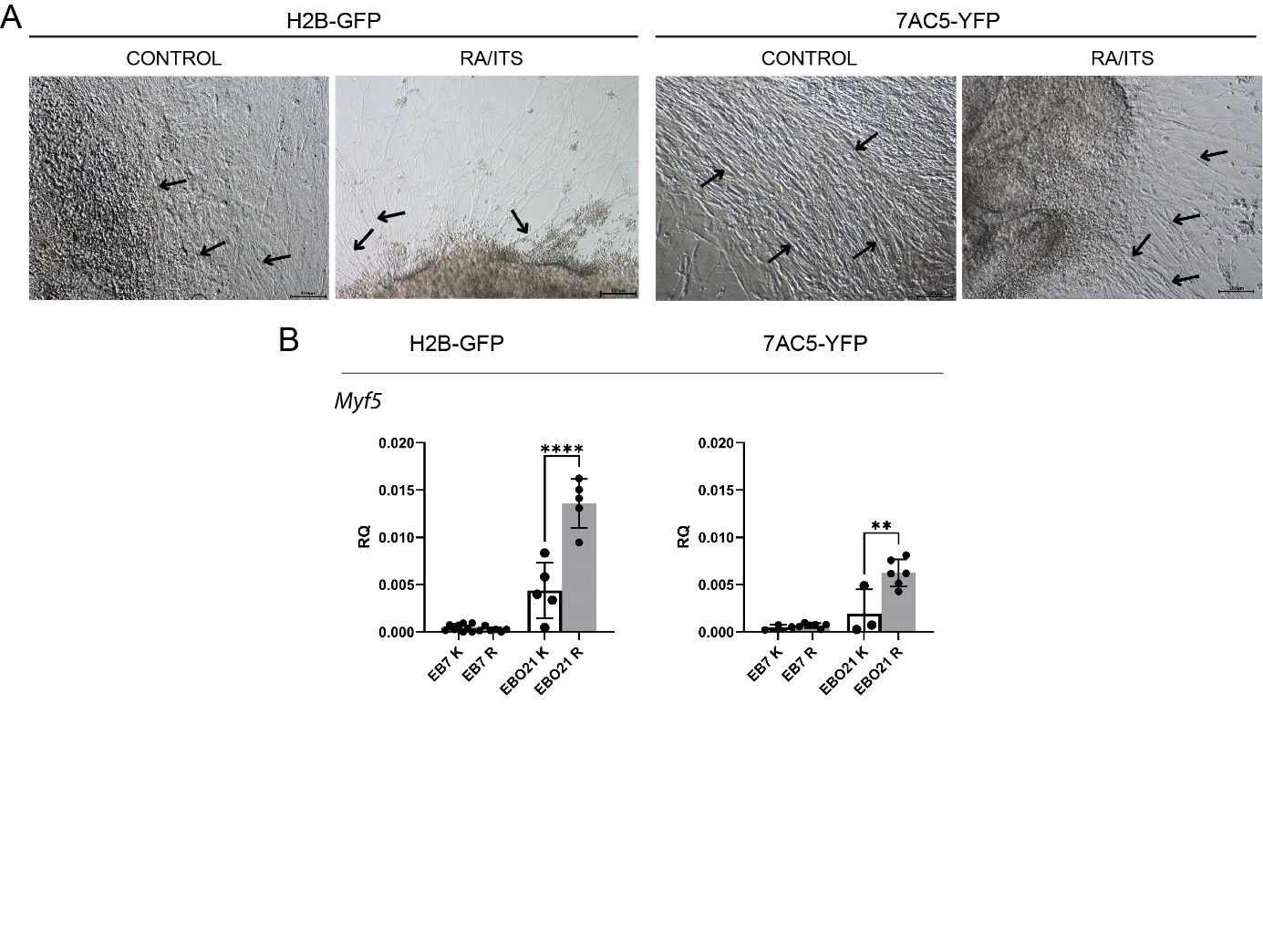


**Figure S4. (A) Expression of selected mesodermal and myogenic markers in H2B-GFP and 7AC5-YFP ESCs treated with either *miR145* or *miR181* or MOCK cells, cultured in RA/ITS medium and analyzed at indicated time-points. (B, C) Analysis of fusion in the co-cultures of C2C12 myoblasts with MOCK or miRNA treated ESCs.** (A) Empty columns refer to MOCK cells, black columns to *miR145* treated cells while grey columns to *miR181* treated cells. Mean values and standard deviations as well as values obtained for each biological replicate are shown. Stars refer to significant differences between MOCK cells and cells treated with indicated miRNA, analyzed at the same time-point. Each experiment was performed in 3 replicates. (B) Representative photos of the indicated types of ESC and C2C12 myoblast co-cultures. (C) Fusion index in the co-cultures of C2C12 myoblasts with either MOCK, *miR181* or *miR145* treated ESCs. Mean values and standard deviations for each co-culture type as well as values obtained for each biological replicate are shown. Stars refer to significant differences between co-cultures of C2C12 myoblasts with miRNA treated cells and co-cultures of myoblasts with MOCK ESCs. Each experiment was performed in 9-13 replicates.


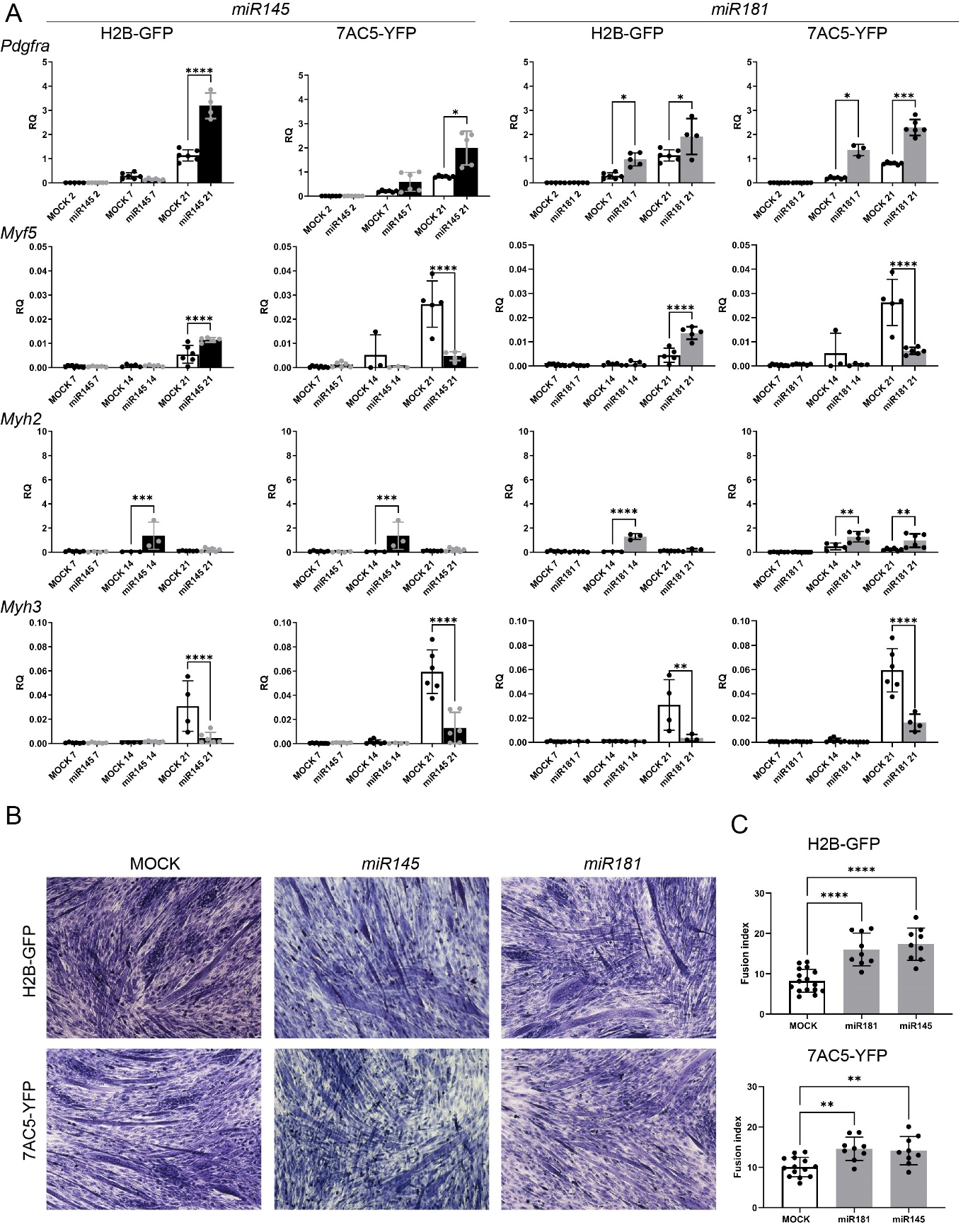


**Figure S5. Expression of selected lncRNAs in H2B-GFP and 7AC5-YFP ESCs treated with either *miR145* or *miR181* or MOCK cells, cultured in RA/ITS medium and analyzed at indicated time-points.** Empty columns refer to MOCK cells, black columns to *miR145* treated cells while grey columns to *miR181* treated cells. The mean values and standard deviation for each sample group as well as values obtained for each biological replicate are shown. Stars refer to all significant differences found between indicated, analyzed samples except ESCs analyzed at the same time-point (MOCK ESCs vs *mi145* treated ESCs, MOCK ESCs vs *mi181* treated ESCs, *mi145* treated ESCs vs *mi181* treated ESCs). *p<0.05, **p<0.01, ***p<0.001, ****p<0.0001. Each experiment was performed in 3 replicates.


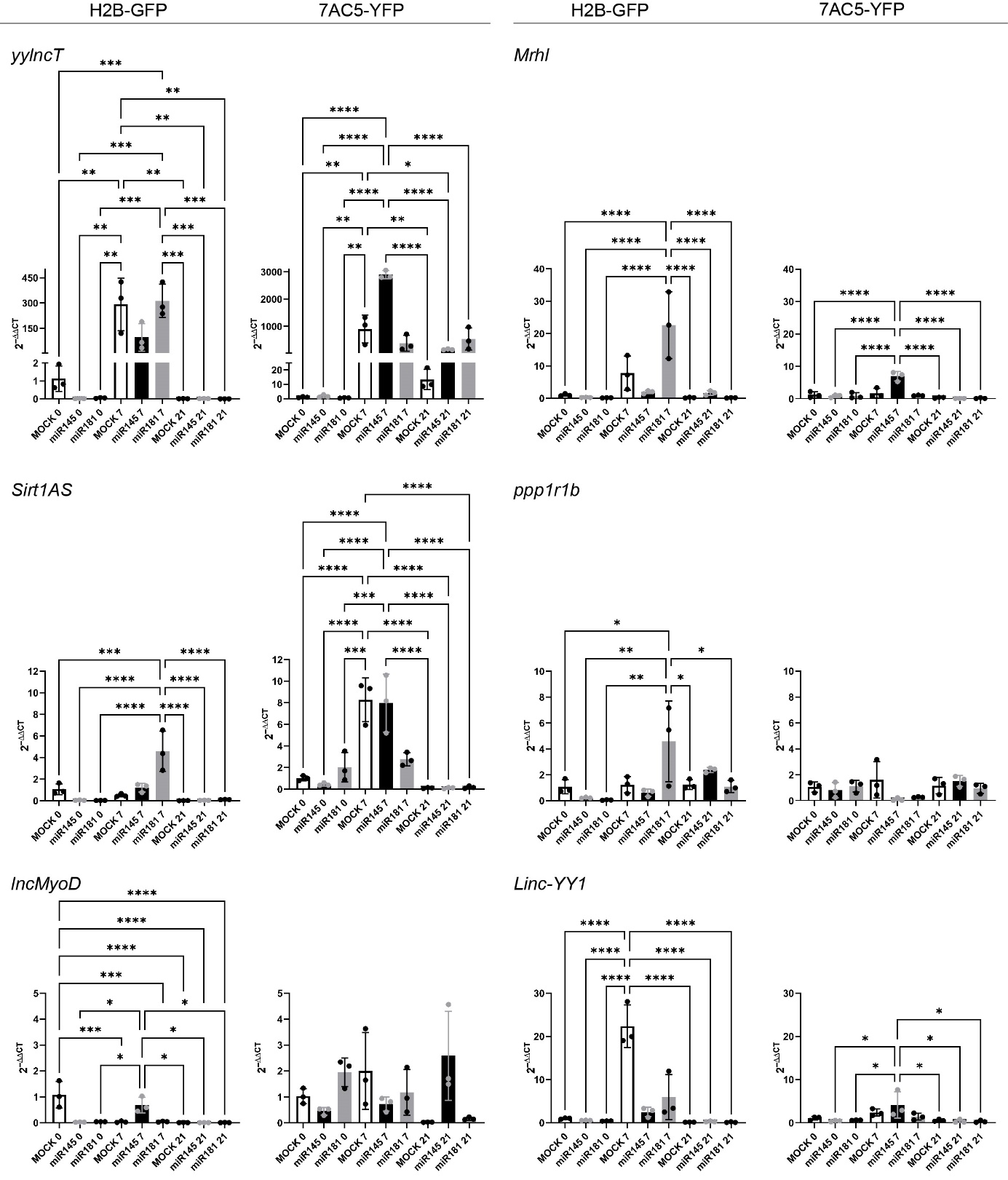


**Figure S6. Expression of selected lncRNAs in H2B-GFP and 7AC5-YFP ESCs treated with either *miR145* or *miR181* or MOCK cells, cultured in RA/ITS medium and analyzed at indicated time-points.** Empty columns refer to MOCK cells, black columns to *miR145* treated cells while grey columns to *miR181* treated cells. The mean values and standard deviation as well as values obtained for each biological replicate are shown. Stars refer to all significant differences found between indicated, analyzed samples except ESCs analyzed at the same time-point (MOCK ESCs vs *mi145* treated ESCs, MOCK ESCs vs *mi181* treated ESCs, *mi145* treated ESCs vs *mi181* treated ESCs). *p<0.05, **p<0.01, ***p<0.001, ****p<0.0001. Each experiment was performed in 3 replicates.


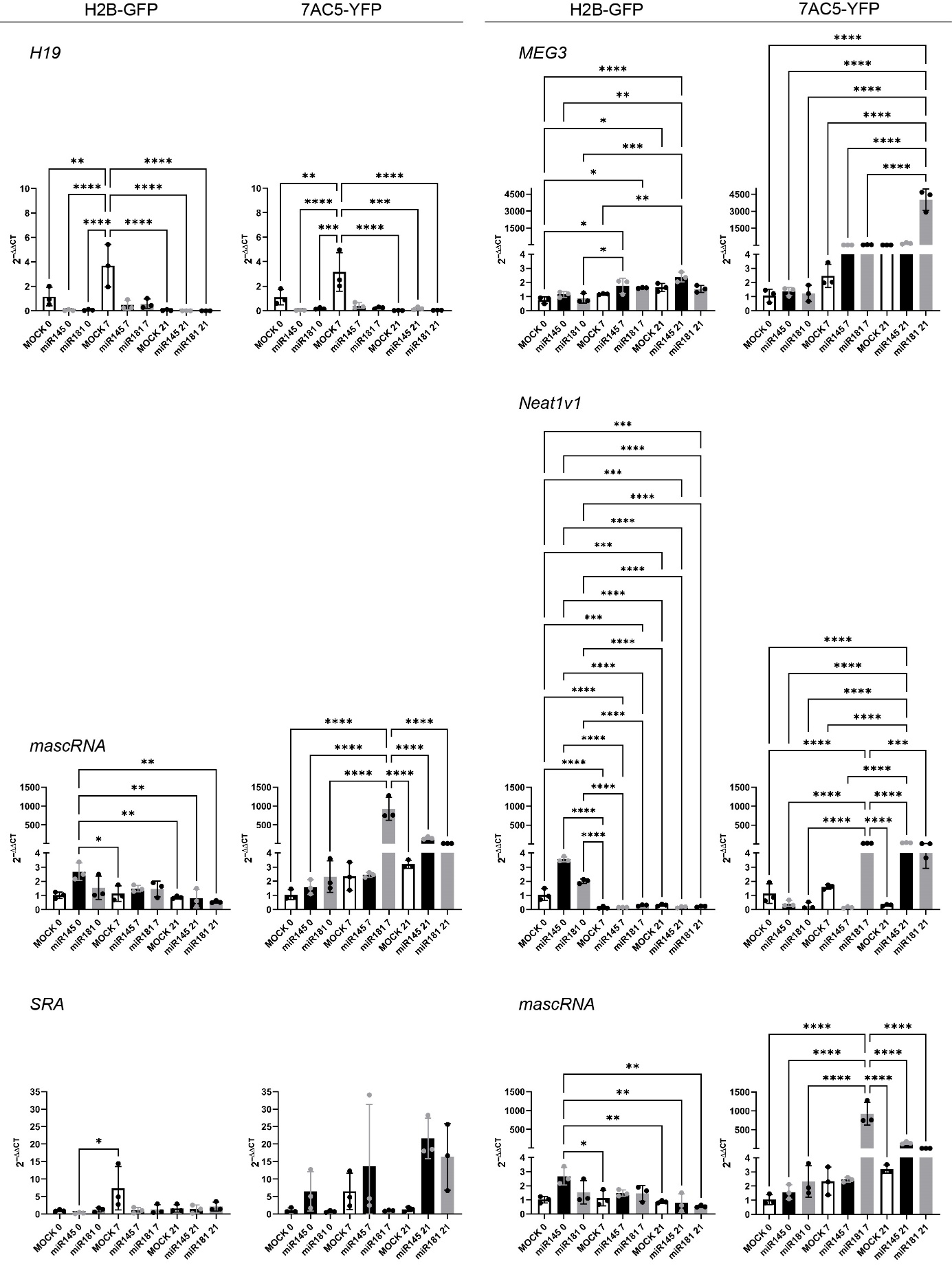


**SUPPLEMENTARY TABLES**

**Table S1. Selected lncRNA participation in myogenesis. Simplified view.**

| **lncRNA** | **ref** | **function** |
| --- | --- | --- |
| *yylncT* | [28] | mesoderm specification |
| *Mrhl* | [29] |  |
| *mascRNA* | [31,33] | proliferation |
| *Sirt1AS* | [34] |  |
| *Malat1* | [30,67] |  |
|  | [64-66] | differentiation |
| *Neat1v1* | [35,36] |  |
| *H19* | [37,39] |  |
| *SRA* | [40,41] |  |
| *lncMyoD* | [41] |  |
| *Linc-MD1* | [43,44] |  |
| *ppp1r1b* | [46,45] |  |
| *Linc-YY1* | [47] |  |
| *MEG3* | [48,49] |  |

**Table S2. ΔCT values for indicated lncRNAs determined in embryo, proliferating (P) and differentiating (D) C2C12 cells, as well as in undifferentiated ESCs of H2B-GFP and 7AC5-YFP cell line.** Bold indicates mean values and standard error of the mean. Below ΔCT from 3 independent experiments are shown.

| **lncRNAs** | **embryo** | **C2C12 P** | **C2C12 D** | **H2B-GFP ESC** | **7AC5-YFP ESC** |
| --- | --- | --- | --- | --- | --- |
| ***yylncT*** | **- 0.88 ± 0.22**  -1.22  -0.48  -0.94 | **5.21 ± 0.35**  5.70  5.40  4.53 | **0** | **7.30 ± 0.32**  7.92  7.12  6.87 | **5.91 ± 0.12**  5.72  5.90  6.12 |
| ***Sirt1AS*** | **0.36 ± 0.2**  0.32  0.37  0.38 | **3.96 ± 0.33**  4.62  3.61  3.66 | **0** | **3.80 ± 0.17**  3.52  4.11  3.86 | **- 0.98 ± 0.12**  - 0.82  - 1.21  - 0.9 |
| ***lncMyoD*** | **1.56 ± 0.22**  1.25  1.99  1.44 | **3.05 ± 0.14**  3.22  2.77  3.15 | **10.64 ± 0.32**  10.18  11.25  10.49 | **4.06 ± 0.07**  4.2  4.0  4.0 | **4.12 ± 0.07**  4.14  4.23  4.0 |
| ***Mrhl*** | **4.28 ± 0.07**  4.35  4.14  4.34 | **4.76 ± 0.24**  5.23  4.47  4.58 | **12.7 ± 0.09**  12.63  12.89  12.59 | **3.42 ± 0.51**  2.41  3.84  4.0 | **5.97 ± 0.25**  6.35  6.06  5.5 |
| ***ppp1r1b*** | **7.02 ± 0.38**  6.77  7.77  6.52 | **10.04 ± 0.43**  10.75  9.27  10.09 | **13.42 ± 0.18**  13.34  13.15  13.76 | **6.38 ± 0.39**  6.25  7.11  5.79 | **8.45 ± 0.19**  8.11  8.76  8.49 |
| ***Linc-YY1*** | **7.52 ± 0.15**  7.78  7.53  7.26 | **5.07 ± 0.21**  5.43  5.09  4.70 | **0** | **6.4 ± 0,26**  6.9  6.0  6.3 | **6.13 ± 0.34**  5.7  6.8  5.9 |
| ***H19*** | **8.75 ± 0.21**  8.59  8.48  9.17 | **10.99 ± 0.06**  10.88  11  11.1 | **11.17 ± 0.05**  11.26  11.13  11.12 | **9.68 ± 0.29**  9.1  9.94  10.01 | **4.71 ± 0.1**  4.9  4.56  4.66 |
| ***mascRNA*** | **8.95 ± 0.1**  8.84  8.87  9.15 | **6.88 ± 0.21**  6.58  6.79  7.28 | **5.49 ± 0.1**  5.68  5.46  5.33 | **4.61 ± 0.29**  4.92  4.04  4.87 | **7.90 ± 0.16**  8.12  7.99  7.6 |
| ***SRA*** | **9.87 ± 0.22**  10.17  9.99  9.45 | **9.47 ± 0.73**  10.65  8.12  9.63 | **8.94 ± 0.12**  8.73  9.14  8.94 | **10.97 ± 0.48**  10.7  10.3  11.9 | **7.5 ± 0.06**  7.5  7.4  7.6 |
| ***MEG3*** | **11.57 ± 0.4**  12.33  10.96  11.43 | **14.54 ± 0.68**  15.74  13.38  14.49 | **13.93 ± 0.37**  14.19  13.19  14.40 | **6.02 ± 0.05**  6.04  5.93  6.09 | **10.3 ± 0.14**  10.43  10.44  10.03 |
| ***Neat1v1*** | **11.63 ± 0.13**  11.87  11.42  11.59 | **11.76 ± 0.56**  12.78  10.84  11.67 | **13.07 ± 0.52**  13.56  13.62  12.02 | **9.15 ± 0.22**  8.75  9.5  9.2 | **14.49 ± 0.33**  13.88  15.01  14.59 |
| ***Malat1*** | **13.47 ± 0.08**  13.59  13.48  13.33 | **15.58 ± 0.48**  16.34  14.76  15.65 | **13.18 ± 0.33**  13.85  12.84  12.86 | **11.43 ± 0.56**  12.48  11.24  10.58 | **13.64 ± 0.68**  14.53  12.31  14.08 |
| ***Linc-MD1*** | **17.50 ± 0.51**  18.24  16.53  17.74 | **0** | **0** | **0** | **0** |

**Table S3. ΔCT mean values and standard error of the mean calculated for indicated lncRNA levels determined in embryo, proliferating (P) and differentiating (D) C2C12 cells, as well as in undifferentiated H2B-GFP and 7AC5-YFP ESC.** The stars refer to significant differences between indicated cell samples in comparison to the lncRNA levels assessed in the embryo E13.5. *p<0.05, **p<0.01, ***p<0.001, ****p<0.0001. Mean ΔCT and standard error of the mean from 3 independent experiments are shown.

| **lncRNAs** | **embryo** | **C2C12 P** | **C2C12 D** | **H2B-GFP ESC** | **7AC5-YFP ESC** |
| --- | --- | --- | --- | --- | --- |
| ***yylncT*** | - 0.88 ± 0.22 | 5.21 ± 0.35  ******** | 0 | 7.30 ± 0.32  ******** | 5.91 ± 0.12  ******** |
| ***Sirt1AS*** | 0.36 ± 0.2 | 3.96 ± 0.33  ******** | 0 | 3.80 ± 0.17  ******** | -0.98 ± 0.12  ****** |
| ***lncMyoD*** | 1.56 ± 0.22 | 3.05 ± 0.14  ****** | 10.64 ± 0.32  ******** | 4.06 ± 0.07  ******** | 4.12 ± 0.07  ******** |
| ***Mrhl*** | 4.28 ± 0.07 | 4.76 ± 0.28 | 12.7 ± 0.09  ******** | 3.42 ± 0.51 | 5.97 ± 0.25  ***** |
| ***ppp1r1b*** | 7.02 ± 0.38 | 10.04 ± 0.43  ******* | 13.42 ± 0.18  ******** | 6.38 ± 0.39 | 8.45 ± 0.19 |
| ***Linc-YY1*** | 7.52 ± 0.15 | 5.07 ± 0.21  ******* | 0 | 6.4 ± 1.13  ***** | 6.13 ± 0.34  ***** |
| ***H19*** | 8.75 ± 0.21 | 10.99 ± 0.06  ******** | 11.17 ± 0.05  ******** | 9.68 ± 0.29  ****** | 4.71 ± 0.1  ******** |
| ***mascRNA*** | 8.95 ± 0.1 | 6.88 ± 0.21  ******** | 5.49 ± 0.1  ******** | 4.61 ± 0.29  ******** | 7.90 ± 0.16  ***** |
| ***SRA*** | 9.87 ± 0.22 | 9.47 ± 0.73 | 8.94 ± 0.12 | 10.97 ± 0.48 | 7.5 ± 0.06  ***** |
| ***MEG3*** | 11.57 ± 0.4 | 14.54 ± 0.68  ****** | 13.93 ± 0.37  ***** | 6.02 ± 0.05  ******** | 10.3 ± 0.14 |
| ***Neat1v1*** | 11.63 ± 0.13 | 11.76 ± 0.56 | 13.07 ± 0.52 | 9.15 ± 0.22  ****** | 14.49 ± 0.33  ****** |
| ***Malat1*** | 13.47 ± 0.08 | 15.58 ± 0.48  ***** | 13.18 ± 0.33 | 11.43 ± 0.56 | 13.64 ±0.68 |
| ***Linc-MD1*** | 17.50±0.51 | 0 | 0 | 0 | 0 |

**Table S4. ΔCT values for indicated lncRNAs determined in undifferentiated H2B-GFP ESCs and differentiated ones, analyzed at the EB7 and EBO21 stage.** K refers to cells cultured in control medium, R – to RA/ITS treated cells. Bold indicates mean ΔCT and standard error of the mean. Below ΔCT from 3 independent experiments are shown.

| **lncRNA** | **ESC**  **H2B-GFP** | **EB7**  **H2B-GFP K** | **EB7**  **H2B-GFP R** | **EBO21**  **H2B-GFP K** | **EBO21**  **H2B-GFP R** |
| --- | --- | --- | --- | --- | --- |
| ***yylncT*** | **7.30 ± 0.32**  7.92  7.12  6.87 | **13.19 ± 0.2**  12.81  13.47  13.29 | **5.36 ± 0.22**  5.42  5.7  4.96 | **9.80 ± 0.31**  10.40  9.40  9.60 | **16.39 ± 0.91**  15.89  18.15  15.13 |
| ***Sirt1AS*** | **3.80 ± 0.17**  3.52  4.11  3.86 | **15.60 ± 0.65**  15.23  16.87  14.7 | **15.71 ± 0.56**  16.82  14.99  15.32 | **17.77 ± 0.33**  18.32  17.81  17.18 | **18.21 ± 0.66**  16.98  18.42  19.23 |
| ***lncMyoD*** | **4.06 ± 0.07**  4.2  4.0  4.0 | **4.72 ± 0.26**  4.97  4.20  5.00 | **8.64 ± 0.68**  7.82  8.11  9.98 | **6.59 ± 0.48**  5.70  7.33  6.75 | **13.98 ± 0.14**  13.77  13.91  14.25 |
| ***Mrhl*** | **3.42 ± 0.18**  2.41  3.84  4.0 | **1.55 ± 0.25**  1.69  1.06  1.89 | **7.43 ± 0.45**  7.57  6.59  8.14 | **2.57 ± 0.08**  2.57  2.71  2.44 | **3.9 ± 0.44**  4.44  4.23  3.04 |
| ***ppp1r1b*** | **6.38 ± 0.39**  6.25  7.11  5.79 | **6.44 ± 0.11**  6.64  6.43  6.26 | **9.69 ± 0.4**  9.34  9.25  10.49 | **10.92 ± 0.23**  11.01  10.48  11.26 | **7.73 ± 0.49**  7.38  8.71  7.11 |
| ***Linc-YY1*** | **6.4 ± 0.27**  6.9  6.0  6.3 | **12.87 ± 1.18**  10.51  14.09  14.02 | **3.59 ± 0.71**  3.65  4.80  2.33 | **9.38 ± 0.37**  9.99  9.45  8.71 | **4.66 ± 0.25**  5.09  4.23  4.67 |
| ***H19*** | **9.68 ± 0.29**  9.1  9.94  10.01 | **17.64 ± 1.25**  17.70  15.44  19.77 | **12.01 ± 0.97**  13.85  11.62  10.57 | **13.54 ± 0.78**  12.03  13.94  14.65 | **8.77 ± 0.62**  9.94  8.56  7.81 |
| ***mascRNA*** | **4.61 ± 0.29**  4.92  4.04  4.87 | **6.72 ± 0.29**  6.23  7.23  6.7 | **8.69 ± 0.33**  9.33  8.51  8.22 | **7.29 ± 0.35**  6.61  7.79  7.48 | **9.58 ± 0.23**  9.13  9.72  9.89 |
| ***SRA*** | **10.97 ± 0.48**  10.7  10.3  11.9 | **15.61 ± 0.57**  14.80  16.72  15.32 | **9.17 ± 0.28**  9.24  8.65  9.62 | **11.94 ± 0.3**  12.05  12.86  11.9 | **9.57 ± 0.33**  10.2  9.42  9.1 |
| ***MEG3*** | **6.02 ± 0.05**  6.04  5.93  6.09 | **11.52 ± 0.4**  12.31  11.22  11.04 | **9.70 ± 0.69**  9.92  10.76  8.41 | **10.00 ± 0.18**  10.26  10.1  9.65 | **10.58 ± 0.21**  10.98  10.46  10.3 |
| ***Neat1v1*** | **9.15 ± 0.22**  8.75  9.5  9.2 | **17.58 ± 0.57**  16.53  18.48  17.73 | **15.83 ± 0.37**  16.05  15.01  14.89 | **17.26 ± 0.63**  18.22  16.07  17.5 | **18.11 ± 0.27**  18.5  17.6  18.23 |
| ***Malat1*** | **11.43 ± 0.56**  12.48  11.24  10.58 | **17.85 ± 0.16**  17.79  17.6  18.16 | **20.42 ± 0.52**  19.65  21.4  20.2 | **14.69 ± 0.37**  14.82  15.26  14.0 | **17.73 ± 0.35**  18.39  17.22  17.59 |
| ***Linc-MD1*** | **0** | **0** | **0** | **0** | **0** |

**Table S5. ΔCT mean values and standard error of the mean calculated for indicated lncRNA levels determined in undifferentiated H2B-GFP ESCs, and differentiated ones, analyzed at EB7 and EBO21 stage.** K refers to cells cultured in control medium, R – to RA/ITS treated cells. Stars refer to significant differences between indicated cell samples in comparison to the lncRNA levels assessed in undifferentiated ESCs. *p<0.05, **p<0.01,
***p< 0.001, ****p<0.0001. Mean ΔCT and standard error of the mean from 3 independent experiments are shown.

| **lncRNAs** | **ESC**  **H2B-GFP** | **EB7**  **H2B- GFP K** | **EB7**  **H2B- GFP R** | **EBO21**  **H2B- GFP K** | **EBO21**  **H2B- GFP R** |
| --- | --- | --- | --- | --- | --- |
| ***yylncT*** | 7.30 ± 0.32 | 13.19 ± 0.2  **** | 5.36 ± 0.22 | 9.80 ± 0.31  * | 16.39 ± 0.91  **** |
| ***Sirt1AS*** | 3.80 ± 0.17 | 15.60 ± 0.65  **** | 15.71 **±** 0.56  **** | 17.77 ± 0.33  **** | 18.21 ± 0.66  **** |
| ***lncMyoD*** | 4.06 ± 0.07 | 4.72 ± 0.26 | 8.64 ± 0.68  **** | 6.59 ± 0.48  ** | 13.98 ± 0.14  **** |
| ***Mrhl*** | 3.42 ± 0.18 | 1.55 ± 0.25  ** | 7.43 ± 0.45  **** | 2.57 ± 0.08 | 3.9 ± 0.44 |
| ***ppp1r1b*** | 6.38 ± 0.39 | 6.44 ± 0.11 | 9.69 ± 0.4  *** | 10.92 ± 0.23  **** | 7.73 ± 0.49 |
| ***Linc-YY1*** | 6.4 ± 0.27 | 12.87 ± 1.18 | 3.59 ± 0.71 | 9.38 ± 0.37 | 4.66 ± 0.25 |
| ***H19*** | 9.68 ± 0.29 | 17.64 ± 1.25  *** | 12.01 ± 0.97 | 13.54 ± 0.78 | 8.77 ± 0.62 |
| ***mascRNA*** | 4.61 ± 0.29 | 6.72 ± 0.29  ** | 8.69 ± 0.33  **** | 7.29 ± 0.35  *** | 9.58 ± 0.23  **** |
| ***SRA*** | 10.97 ± 0.48 | 15.61 ± 0.57  **** | 9.17 ± 0.28 | 11.94 ± 0.3 | 9.57 ± 0.33 |
| ***MEG3*** | 6.02 ± 0.05 | 11.52 ± 0.4  **** | 9.70 ± 0.69  *** | 10.00 ± 0.18  *** | 10.58 ± 0.21  **** |
| ***Neat1v1*** | 9.15 ± 0.22 | 17.58 ± 0.57  **** | 15.83 ± 0.37  ****** | 17.26 ± 0.63  **** | 18.11 ± 0.27  ****** |
| ***Malat1*** | 11.43 ± 0.56 | 17.85 ± 0.16  **** | 20.42 ± 0.52  ****** | 14.69 ± 0.37  ** | 17.73 ± 0.35  ****** |
| ***Linc-MD1*** | 0 | 0 | 0 | 0 | 0 |

**Table S6. ΔCT values for indicated lncRNAs determined in undifferentiated 7AC5-YFP ESCs and differentiated ones, analyzed at EB7 and EBO21 stage.** K refers to cells cultured in control medium, R – to RA/ITS treated cells. Bold refers to mean ΔCT and standard error of the mean. Below ΔCT from 3 independent experiments are shown.

| **lncRNA** | **ESC**  **7AC5-YFP** | **EB7**  **7AC5-YFP K** | **EB7**  **7AC5-YFP R** | **EBO21**  **7AC5-YFP K** | **EBO21**  **7AC5-YFP R** |
| --- | --- | --- | --- | --- | --- |
| ***yylncT*** | **5.91 ± 0.12**  5.72  5.90  6.12 | **12.27 ± 0.68**  13.56  11.98  11.27 | **4.51 ± 0.13**  4.72  4.53  4.28 | **6.95 ± 0.22**  6.52  7.21  7.12 | **17.88 ± 0.44**  18.65  17.85  17.14 |
| ***Sirt1AS*** | **-0.98 ± 0.12**  -0.82  -1.21  -0.9 | **12.02 ± 0.83**  11.08  11.93  13.05 | **13.25 ± 0.31**  12.81  13.86  13.08 | **16.19 ± 0.71**  17.01  16.78  14.78 | **17.21 ± 0.92**  18.61  17.53  15.49 |
| ***lncMyoD*** | **4.12 ± 0.07**  4.14  4.23  4.0 | **4.59 ± 0.24**  4.96  4.15  4.65 | **13.29 ± 0.74**  14.77  12.66  12.45 | **3.82 ± 0.27**  4.35  3.47  3.65 | **3.89 ± 0.13**  3.83  4.13  3.70 |
| ***Mrhl*** | **5.97 ± 0.25**  6.35  6.06  5.5 | **3.74 ± 0.04**  3.76  3.8  3.67 | **2.34 ± 0.15**  2.64  2.17  2.21 | **1.98 ± 0.12**  1.96  2.21  1.78 | **4.94 ± 0.14**  4.78  5.21  4.82 |
| ***ppp1r1b*** | **8.45 ± 0.19**  8.11  8.76  8.49 | **5.36 ± 0.46**  5.67  4.46  5.95 | **8.36 ± 0.38**  7.83  8.15  9.09 | **6.49 ± 0.11**  6.63  6.27  6.58 | **11.03 ± 0.86**  11.59  12.18  9.31 |
| ***Linc-YY1*** | **6.13 ± 0.34**  5.7  6.8  5.9 | **12.32 ± 0.35**  12.00  11.94  13.02 | **2.83 ± 0.56**  2.47  3.94  2.09 | **8.33 ± 0.35**  8.18  7.82  8.99 | **4.50 ± 0.21**  4.09  4.72  4.70 |
| ***H19*** | **4.71 ± 0.1**  4.9  4.56  4.66 | **14.68 ± 0.55**  15.64  13.74  14.66 | **9.80 ± 0.29**  9.24  10.24  9.91 | **8.94 ± 0.89**  10.21  7.23  9.38 | **3.91 ± 0.31**  4.53  3.54  3.65 |
| ***mascRNA*** | **7.90 ± 0.16**  8.12  7.99  7.6 | **10.72 ± 0.67**  9.61  11.92  10.63 | **12.64 ± 0.64**  11.2  12.96  13.24 | **9.7 ± 0.52**  9.53  10.68  8.9 | **9.76 ± 0.28**  9.34  9.64  10.30 |
| ***SRA*** | **7.5 ± 0.06**  7.5  7.4  7.6 | **14.45 ± 0.45**  15.16  13.61  14.58 | **6.61 ± 0.29**  7.18  6.46  6.2 | **11.3 ± 0.66**  10.03  11.64  12.23 | **9.13 ± 0.29**  8.69  9.03  9.67 |
| ***MEG3*** | **10.3 ± 0.14**  10.43  10.44  10.03 | **14.55 ± 0.57**  14.02  12.84  14.8 | **14.75 ± 0.52**  13.76  14.96  15.52 | **12.64 ± 0.2**  12.12  12.81  12.45 | **13.38 ± 0.22**  13.1  13.23  13.81 |
| ***Neat1v1*** | **14.49 ± 0.33**  13.88  15.01  14.59 | **15.80 ± 0.49**  14.85  16.07  16.47 | **21.48 ± 0.37**  21.37  22.17  20.89 | **17.63 ± 0.59**  18.53  17.85  16.52 | **17.21 ± 0.26**  17.03  17.73  16.87 |
| ***Malat1*** | **13.64 ± 0.68**  14.53  12.31  14.08 | **18.15 ± 0.51**  18.49  18.83  17.14 | **16.91 ± 0.45**  16.43  17.82  16.49 | **14.45 ± 0.19**  14.75  14.49  14.1 | **11.24 ± 0.36**  11.9  11.16  10.65 |
| ***Linc-MD1*** | **0** | **0** | **0** | **0** | **0** |

**Table S7. ΔCT mean values and standard error of the mean calculated for indicated lncRNA levels determined in undifferentiated 7AC5-YFP ESCs and differentiated ones, analyzed at EB7 and EBO21 stage.** K refers to cells cultured in control medium, R – to RA/ITS treated cells. Stars refer to significant differences between indicated cell samples in comparison to the lncRNA levels assessed in undifferentiated ESCs. *p<0.05, **p<0.01, ***p<0.001, ****p<0.0001. Mean ΔCT and standard error of the mean from 3 independent experiments are shown. B

| **lncRNAs** | **ESC**  **7AC5-YFP** | **EB7**  **7AC5-YFP K** | **EB7**  **7AC5-YFP R** | **EBO21**  **7AC5-YFP K** | **EBO21**  **7AC5-YFP R** |
| --- | --- | --- | --- | --- | --- |
| ***yylncT*** | 5.91 ± 0.12 | 12.27 ± 0.68  **** | 4.51 ± 0.13 | 6.95 ± 0.22 | 17.88 ± 0.44  **** |
| ***Sirt1AS*** | -0.98 ± 0.12 | 12.02 ± 0.83  **** | 13.25 ± 0.31  **** | 16.19 ± 0.71  **** | 17.21 ± 0.92  **** |
| ***lncMyoD*** | 4.12 ± 0.07 | 4.59 ± 0.24 | 13.29 ± 0.74  **** | 3.82 ± 0.27 | 3.89 ± 0.13 |
| ***Mrhl*** | 5.97 ± 0.25 | 3.74 ± 0.04  **** | 2.34 ± 0.15  **** | 1.98 ± 0.12  **** | 4.94 ± 0.14  ** |
| ***ppp1r1b*** | 8.45 ± 0.19 | 5.36 ± 0.46  ** | 8.36 ± 0.38 | 6.49 ± 0.11 | 11.03 ± 0.86  * |
| ***Linc-YY1*** | 6.13 ± 0.34 | 12.32 ± 0.35  **** | 2.83 ± 0.56  *** | 8.33 ± 0.35  * | 4.50 ± 0.21 |
| ***H19*** | 4.71 ± 0.1 | 14.68 ± 0.55  **** | 9.80 ± 0.29  *** | 8.94 ± 0.89  ** | 3.91 ± 0.31 |
| ***mascRNA*** | 7.90 ± 0.16 | 10.72 ± 0.67  * | 12.64 ± 0.64  *** | 9.7 ± 0.52 | 9.76 ± 0.28 |
| ***SRA*** | 7.5 ± 0.06 | 14.45 ± 0.45  **** | 6.61 ± 0.29 | 11.3 ± 0.66  *** | 9.13 ± 0.29 |
| ***MEG3*** | 10.3 ± 0.14 | 14.55 ± 0.57 | 14.75 ± 0.52  * | 12.64 ± 0.2 | 13.38 ± 0.22 |
| ***Neat1v1*** | 14.49 ± 0.33 | 15.80 ± 0.49 | 21.48 ± 0.37 ****** | 17.63 ± 0.59  ** | 17.21 ± 0.26  **** |
| ***Malat1*** | 13.64 ± 0.68 | 18.15 ± 0.51  *** | 16.91 ± 0.45  **** | 14.45 ± 0.19 | 11.24 ± 0.36  *** |
| ***Linc-MD1*** | 0 | 0 | 0 | 0 | 0 |

**Table S8. ΔCT mean values and standard error of the mean calculated for indicated lncRNA levels determined in H2B-GFP ESCs, analyzed at 0, 7, and 21 days after miRNA overexpression.** Stars refer to significant differences between indicated cell samples in comparison to the lncRNA levels assessed in undifferentiated ESCs. *p<0.05, **p<0.01, ***p<0.001, ****p<0.0001. Mean ΔCT and standard error of the mean from 3 independent experiments are shown.

| **lncRNAs** | **MOCK**  **0** | **miR145 0** | **miR181 0** | **MOCK**  **7** | **miR145 7** | **miR181 7** | **MOCK**  **21** | **miR145 21** | **miR181 21** |
| --- | --- | --- | --- | --- | --- | --- | --- | --- | --- |
| ***yylncT*** | 14.57 ± 0.49  **** | 20.33 ± 0.26  **** | 19.00 ± 0.34  **** | 6.57 ± 0.56 | 8.34 ± 0.72 | 6.32 ± 0.25 | 21.11 ± 0.43  **** | 20.59 ± 0.18  **** | 23.84 ± 0.52  **** |
| ***Sirt1AS*** | 4.68 ± 0.37 | 9.15 ± 0.35  **** | 10.15 ± 0.18  **** | 5.71 ± 0.22  ** | 4.46 ± 0.3 | 2.56 ± 0.35 | 11.04 ± 0.35  **** | 9.55 ± 0.22  **** | 7.87 ± 0.12  **** |
| ***lncMyoD*** | 4.38 ± 0.41 | 9.85 ± 0.24  **** | 9.02 ± 0.04  **** | 9.36 ± 0.6  **** | 5.01 ± 0.35 | 9.1 ± 0.46  **** | 11.26 ± 0.26  **** | 12.58 ± 0.44  **** | 11.22 ± 0.3  **** |
| ***Mrhl*** | 9.4 ± 0.27  **** | 11.35 ± 0.08  **** | 12.11 ± 0.13  **** | 6.71 ± 0.66  *** | 8.49 ± 0.22  **** | 5.01 ± 0.41 | 11.90 ± 0.5  **** | 8.75 ± 0.37  **** | 12.27 ± 0.4  **** |
| ***ppp1r1b*** | 23.92 ± 0.39  **** | 26.15 ± 0.18  **** | 28.25 ± 0.35  **** | 23.78 ± 0.51  **** | 24.74 ± 0.46  **** | 22.09 ± 0.83  **** | 23.64 ± 0.24  **** | 22.67 ± 0.07  **** | 23.52 ± 0.21  **** |
| ***Linc-YY1*** | 13.65 ±0.12  **** | 14.51 ± 0.09  **** | 14.96 ± 0.03  **** | 9.19 ± 0.18  *** | 12.47 ± 0.46  **** | 11.43 ± 0.69  **** | 16.54 ± 0.2  **** | 15.55 ± 0.3  **** | 16.73 ± 0.45  **** |
| ***H19*** | 6.36 ± 0.56  ** | 11.02 ± 0.71 | 10.47 ± 0.58 | 4.59 ± 0.43  **** | 7.76 ± 0.65 | 7.35 ± 0.54 | 11.05 ± 0.71 | 14.94 ± 0.05  **** | 14.34 ± 0.34  *** |
| ***mascRNA*** | 12.84± 0,2   \|  \| \| --- \|   **** | 11.44 ± 0.19  **** | 12.34 ± 0.41  **** | 12.77 ± 0.4  **** | 12.28 ± 0.13  **** | 12.37 ± 0.36  **** | 13.06 ± 0.1  **** | 13.59 ± 0.8  **** | 13.72 ± 0.12  **** |
| ***SRA*** | 9.98 ± 0.21 | 11.88 ± 0.5 | 9.83 ± 0.52 | 7.44 ± 0.7  * | 10.25 ± 0.81 | 10.05 ± 0.83 | 9.47 ± 0.51 | 10.01 ± 1 | 9.05 ± 0.46 |
| ***MEG3*** | 16.36 ± 0.17  **** | 16.17 ± 0.15  **** | 16.6 ± 0.29  **** | 16.12 ± 0.03  **** | 15.59 ± 0.28  **** | 15.67 ± 0.01  **** | 15.66 ± 0.15  **** | 15.12 ± 0.12  **** | 15.66 ± 0.12  **** |
| ***Neat1v1*** | 14.05 ± 0.31  **** | 12.22 ± 0.04  **** | 13.05 ± 0.07  **** | 17.05 ± 0.45  **** | 16.79 ± 0.03  **** | 15.81 ± 0.14  **** | 15.74 ± 0.17  **** | 16.66 ± 0.29  **** | 16.25 ± 0.18  **** |
| ***Malat1*** | 14.19 ± 0.02  **** | 13.2 ± 0.03  ** | 13.79 ± 0.13  **** | 13.89 ± 0.11  **** | 12.89 ± 0.06  * | 14.21 ± 0.36  **** | 13.3 ± 0.21  *** | 12.62 ± 0.04 | 12.81 ± 0.28  * |

**Table S9. ΔCT values for indicated lncRNAs determined in H2B-GFP ESCs, analyzed at 0, 7, and 21 days after miRNA overexpression.** Bold refers to mean ΔCT and standard error of the mean. Below ΔCT from 3 independent experiments are shown.

| **lncRNAs** | **MOCK**  **0** | **miR145 0** | **miR181 0** | **MOCK**  **7** | **miR145 7** | **miR181 7** | **MOCK**  **21** | **miR145 21** | **miR181 21** |
| --- | --- | --- | --- | --- | --- | --- | --- | --- | --- |
| ***yylncT*** | **14.57 ± 0.49**  13.62  14.86  15.25 | **20.33 ± 0.26**  19.82  20.48  20.70 | **19.00 ± 0.34**  18.47  18.90  19.64 | **6.57 ± 0.56**  6.21  5.84  7.66 | **8.34 ± 0.72**  7.01  8.51  9.5 | **6.32 ± 0.25**  6.68  6.46  5.84 | **21.11 ± 0.43**  20.31  21.25  21.78 | **20.59 ± 0.18**  20.64  20.26  20.89 | **23.84 ± 0.52**  24.81  23.01  23.71 |
| ***Sirt1AS*** | **4.68 ± 0.37**  4.89  5.21  3.96 | **9.15 ± 0.35**  8.46  9.46  9.55 | **10.15 ± 0.18**  10.43  10.2  9.81 | **5.71 ± 0.22**  5.9  5.97  5.27 | **4.46 ± 0.3**  5.07  4.23  4.1 | **2.56 ± 0.35**  1.97  2.55  3.18 | **11.04 ± 0.35**  10.71  11.73  10.68 | **9.55 ± 0.22**  9.13  9.87  9.67 | **7.87 ± 0.12**  8.06  7.91  7.65 |
| ***lncMyoD*** | **4.38 ± 0.41**  4.36  5.10  3.68 | **9.85 ± 0.24**  9.39  10.21  9.95 | **9.02 ± 0.04**  9.10  9.01  8.97 | **9.36 ± 0.6**  10.32  8.26  9.51 | **5.01 ± 0.35**  5.15  4.34  5.54 | **9.1 ± 0.46**  8.97  8.37  9.96 | **11.26 ± 0.26**  11.30  11.69  10.80 | **12.58 ± 0.44**  12.83  13.18  11.73 | **11.22 ± 0.3**  11.50  10.62  11.55 |
| ***Mrhl*** | **9.4 ± 0.27**  9.54  9.79  8.87 | **11.35 ± 0.08**  11.41  11.19  11.45 | **12.11 ± 0.13**  12.15  12.33  11.87 | **6.71 ± 0.66**  6.55  5.67  7.93 | **8.49 ± 0.22**  8.92  8.39  8.18 | **5.01 ± 0.41**  4.9  5.78  4.36 | **11.90 ± 0.5**  11.10  11.79  12.83 | **8.75 ± 0.37**  8.62  8.18  9.45 | **12.27 ± 0.4**  12.26  11.59  12.98 |
| ***ppp1r1b*** | **23.92 ± 0.39**  24.37  24.24  23.15 | **26.15 ± 0.18**  26.22  25.81  26.43 | **28.25 ± 0.35**  28.11  28.91  27.73 | **23.78 ± 0.51**  23.03  23.56  24.75 | **24.74 ± 0.46**  24.20  25.65  24.37 | **22.09 ± 0.83**  21.08  21.47  23.74 | **23.64 ± 0.24**  23.95  23.17  23.80 | **22.67 ± 0.07**  22.81  22.58  22.63 | **23.52 ± 0.21**  23.41  23.93  23.24 |
| ***Linc-YY1*** | **13.65 ±0.12**  13.55  13.89  13.51 | **14.51 ± 0.09**  14.34  14.55  14.65 | **14.96 ± 0.03**  15.01  14.92  14.95 | **9.19 ± 0.18**  9.33  8.84  9.40 | **12.47 ± 0.46**  12.13  11.92  13.38 | **11.43 ± 0.69**  10.07  11.90  12.33 | **16.54 ± 0.2**  16.18  16.58  16.88 | **15.55 ± 0.3**  14.97  15.94  15.75 | **16.73 ± 0.45**  15.87  17.29  17.13 |
| ***mascRNA*** | **12.84± 0,2**   \| 12.78  12.53  13.2 \| \| --- \| | **11.44 ± 0.19**  11.72  11.52  11.08 | **12.34 ± 0.41**  12.81  12.69  11.52 | **12.77 ± 0.4**  12.9  13.38  12.03 | **12.28 ± 0.13**  12.04  12.32  12.47 | **12.37 ± 0.36**  12.12  11.91  13.09 | **13.06 ± 0.1**  13.08  12.89  13.22 | **13.59 ± 0.8**  12.23  13.55  14.98 | **13.72 ± 0.12**  13.87  13.8  13.49 |
| ***H19*** | **6.36 ± 0.56**  7.28  5.35  6.45 | **11.02 ± 0.71**  11.33  12.07  9.67 | **10.47 ± 0.58**  9.52  10.39  11.52 | **4.59 ± 0.43**  3.92  5.39  4.48 | **7.76 ± 0.65**  8.39  8.44  6.47 | **7.35 ± 0.54**  7.54  8.18  6.33 | **11.05 ± 0.71**  11.84  11.68  9.63 | **14.94 ± 0.05**  14.97  14.85  15.02 | **14.34 ± 0.34**  14.76  14.61  13.66 |
| ***SRA*** | **9.98 ± 0.21**  10.19  10.23  9.57 | **11.88 ± 0.5**  11.07  11.80  12.78 | **9.83 ± 0.52**  10.85  9.50  9.15 | **7.44 ± 0.7**  8.51  7.68  6.13 | **10.25 ± 0.81**  9.91  9.05  11.8 | **10.05 ± 0.83**  11.22  10.50  8.45 | **9.47 ± 0.51**  10.03  8.45  9.95 | **10.01 ± 1**  8.62  9.45  11.97 | **9.05 ± 0.46**  9.48  9.56  8.13 |
| ***MEG3*** | **16.36 ± 0.17**  16.49  16.57  16.03 | **16.17 ± 0.15**  16.45  16.09  15.96 | **16.6 ± 0.29**  16.87  16.91  16.02 | **16.12 ± 0.03**  16.08  16.12  16.17 | **15.59 ± 0.28**  16.15  15.33  15.3 | **15.67 ± 0.01**  15.65  15.67  15.7 | **15.66 ± 0.15**  15.92  15.65  15.41 | **15.12 ± 0.12**  15.13  14.91  15.33 | **15.66 ± 0.12**  15.42  15.74  15.52 |
| ***Neat1v1*** | **14.05 ± 0.31**  13.46  14.55  14.14 | **12.22 ± 0.04**  12.14  12.26  12.27 | **13.05 ± 0.07**  13.18  12.96  13.02 | **17.05 ± 0.45**  17.56  17.44  16.16 | **16.79 ± 0.03**  16.84  16.79  16.74 | **15.81 ± 0.14**  15.73  15.62  15.07 | **15.74 ± 0.17**  16.05  15.71  15.46 | **16.66 ± 0.29**  16.31  16.42  17.24 | **16.25 ± 0.18**  16.61  16.03  16.1 |
| ***Malat1*** | **14.19 ± 0.02**  14.16  14.17  14.23 | **13.2 ± 0.03**  13.14  13.23  13.23 | **13.79 ± 0.13**  13.71  14.05  13.62 | **13.89 ± 0.11**  13.67  14.01  13.99 | **12.89 ± 0.06**  12.9  12.79  12.99 | **14.21 ± 0.36**  13.48  14.59  14.55 | **13.3 ± 0.21**  13.63  12.91  13.37 | **12.62 ± 0.04**  12.61  12.57  12.69 | **12.81 ± 0.28**  13.36  12.5  12.57 |

**Table S10. ΔCT mean values and standard error of the mean calculated for indicated lncRNA levels determined in 7AC5-YFP ESCs, analyzed at 0, 7, and 21 days after miRNA overexpression.** Stars refer to significant differences between indicated cell samples in comparison to the lncRNA levels assessed in undifferentiated ESCs. *p<0.05, **p<0.01, ***p<0.001, ****p<0.0001. Mean ΔCT and standard error of the mean from 3 independent experiments are shown.

| **lncRNAs** | **MOCK**  **0** | **miR145 0** | **miR181 0** | **MOCK**  **7** | **miR145 7** | **miR181 7** | **MOCK**  **21** | **miR145 21** | **miR181 21** |
| --- | --- | --- | --- | --- | --- | --- | --- | --- | --- |
| ***yylncT*** | 23.06 ± 0.32  **** | 22.45 ± 0.47  **** | 23.99 ± 0.4  **** | 13.46 ± 0.62  **** | 2.89 ± 0.35  ** | 14.73 ± 0.62  **** | 19.45 ± 0.41  **** | 17.16 ± 0.46  **** | 4.3 ± 0.71 |
| ***Sirt1AS*** | 7.88 ± 0.2  **** | 9.09 ± 0.24  **** | 7.05 ± 0.59  **** | 4.86 ± 0.22  **** | 4.94 ± 0.3  **** | 6.43 ± 0.18  **** | 11.11 ± 0.34  **** | 11.3 ± 0.6  **** | 10.7 ± 0.64  **** |
| ***lncMyoD*** | 8.37 ± 0.24  **** | 9.57 ± 0.3  **** | 7.44 ± 0.26  *** | 7.66 ± 0.67  *** | 8.91 ± 0.36  **** | 8.44 ± 0.68  **** | 13.71 ± 0.21  **** | 7.19 ± 0.51  *** | 11.03 ± 0.27  **** |
| ***Mrhl*** | 10.20 ± 0.68  *** | 10.6 ± 0.53  **** | 10.82 ± 0.82  **** | 9.74 ± 0.67  ** | 7.42 ± 0.19  *** | 10.22 ± 0.08  **** | 11.92 ± 0.5  **** | 13.29 ± 0.07  **** | 12.82 ± 0.59  **** |
| ***ppp1r1b*** | 24.64 ± 0.36  **** | 25.25 ± 0.71  **** | 24.58 ± 0.4  **** | 24.38 ± 0.81  **** | 28.57 ± 0.93  **** | 26.48 ± 0.11  **** | 24.61 ± 0.57  **** | 24.06 ± 0.25  **** | 24.50 ± 0.39  **** |
| ***Linc-YY1*** | 13.79 ± 0.38  **** | 15.14 ± 0.39  **** | 14.49 ± 0.11  **** | 12.58 ± 0.27  **** | 11.97 ± 0.58  **** | 13.09 ± 0.5  **** | 14.68 ± 0.35  **** | 15.66 ± 0.8  **** | 15.78 ± 0.7  **** |
| ***mascRNA*** | 19.2 ± 0.3  **** | 18.59 ± 0.27  **** | 18.08 ± 0.38  **** | 18.06 ± 0.37  **** | 17.91 ± 0.06  **** | 9.39 ± 0.26 | 17.44 ± 0.12  **** | 12.74 ± 0.68  **** | 15.74 ± 0.05  **** |
| ***H19*** | 10.2 ± 0.45  **** | 14.82 ± 0.29  **** | 12.78 ± 0.32  **** | 8.64 ± 0.39  **** | 11.58 ± 0.43  **** | 12.36 ± 0.34  **** | 15.49 ± 0.31  **** | 12.93 ± 0.63  **** | 14.66 ± 0.15  **** |
| ***SRA*** | 11.25 ± 0.46  ** | 8.99 ± 0.84 | 11.75 ± 0.33  ** | 8.87 ± 0.69 | 8.40 ± 1.15 | 11.29 ± 0.13  ** | 10.98 ± 0.47  * | 6.85 ± 0.21 | 7.42 ± 0.57 |
| ***MEG3*** | 25.36 ± 0.42  **** | 24.94 ± 0.19  **** | 25.17 ± 0.42  **** | 24.1 ± 0.26  **** | 22.82 ± 0.19  **** | 20.89 ± 0.93  **** | 22.47 ± 0.17  **** | 18.17 ± 0.36  **** | 13.42 ± 0.22  *** |
| ***Neat1v1*** | 16.98 ± 0.49 | 18.35 ± 0.41  ** | 19.27 ± 0.7  *** | 16.31 ± 0.08 | 19.84 ± 0.3  **** | 12.96 ± 1.3 | 18.48 ± 0.28  ** | 12.07 ± 0.14 | 14.6 ± 0.37 |
| ***Malat1*** | 14.37 ± 0.13 | 14.57 ± 0.36 | 13.3 ± 0.3 | 13,91 ± 0.08 | 13.92 ± 0.24 | 10.71 ± 0.53  ** | 12.98 ± 0.13 | 11.6 ± 0.34  * | 13.54 ± 0.53 |

**Table S11. ΔCT values for indicated lncRNAs determined in 7AC5-YFP ESCs, analyzed at 0, 7, and 21 days after miRNA overexpression.** Bold refers to mean ΔCT and standard error of the mean. Below ΔCT from 3 independent experiments are shown.

| **lncRNAs** | **MOCK**  **0** | **miR145 0** | **miR181 0** | **MOCK**  **7** | **miR145 7** | **miR181 7** | **MOCK**  **21** | **miR145 21** | **miR181 21** |
| --- | --- | --- | --- | --- | --- | --- | --- | --- | --- |
| ***yylncT*** | **23.06 ± 0.32**  23.58  23.13  22.48 | **22.45 ± 0.47**  23.21  22.53  21.60 | **23.99 ± 0.4**  23.91  23.36  24.72 | **13.46 ± 0.62**  13.02  14.69  12.69 | **11.56± 0.35**  11.61  11.48  11.6 | **14.73 ± 0.62**  15.7  13.57  14.92 | **19.45 ± 0.41**  19.95  19.76  18.63 | **17.16 ± 0.46**  17.73  17.51  16.24 | **14.3 ± 0.71**  15.59  14.2  13.13 |
| ***Sirt1AS*** | **7.88 ± 0.2**  8.23  7.86  7.55 | **9.09 ± 0.24**  9.29  9.38  8.61 | **7.05 ± 0.59**  6.06  8.09  7.11 | **4.86 ± 0.22**  4.62  5.31  4.66 | **4.94 ± 0.3**  4.48  4.85  5.5 | **6.43 ± 0.18**  6.1  6.73  6.47 | **11.11 ± 0.34**  10.85  11.78  10.7 | **11.3 ± 0.6**  10.6  12.5  10.8 | **10.7 ± 0.64**  9.6  11.8  10.7 |
| ***lncMyoD*** | **8.37 ± 0.24**  8.79  8.37  7.95 | **9.57 ± 0.3**  9.27  10.18  9.28 | **7.44 ± 0.26**  7.24  7.96  7.14 | **7.66 ± 0.67**  6.51  8.84  7.65 | **8.91 ± 0.36**  8.66  9.62  8.46 | **8.44 ± 0.68**  8.44  9.61  7.27 | **13.71 ± 0.21**  13.36  14.09  13.69 | **7.19 ± 0.51**  7.60  6.18  7.79 | **11.03 ± 0.27**  10.50  11.23  11.36 |
| ***Mrhl*** | **10.20 ± 0.68**  10.15  9.05  11.41 | **10.6 ± 0.53**  10.14  11.66  10.00 | **10.82 ± 0.82**  11.86  11.39  9.21 | **9.74 ± 0.67**  8.43  10.57  10.24 | **7.42 ± 0.19**  7.79  7.18  7.3 | **10.22 ± 0.08**  10.33  10.28  10.06 | **11.92 ± 0.5**  11.52  12.92  11.33 | **13.29 ± 0.07**  13.16  13.39  13.32 | **12.82 ± 0.59**  13.18  13.63  11.67 |
| ***ppp1r1b*** | **24.64 ± 0.36**  24.17  24.41  25.35 | **25.25 ± 0.71**  26.56  25.08  24.12 | **24.58 ± 0.4**  24.28  24.08  25.38 | **24.38 ± 0.81**  25.79  22.97  24.39 | **28.57 ± 0.93**  29.38  29.61  26.72 | **26.48 ± 0.11**  26.67  26.28  26.51 | **24.61 ± 0.57**  23.87  25.73  24.24 | **24.06 ± 0.25**  23.73  23.91  24.56 | **24.50 ± 0.39**  25.59  24.50  24.34 |
| ***Linc-YY1*** | **13.79 ± 0.38**  13.44  13.39  14.55 | **15.14 ± 0.39**  14.99  15.88  14.55 | **14.49 ± 0.11**  14.43  14.34  14.70 | **12.58 ± 0.27**  12.67  12.07  13.01 | **11.97 ± 0.58**  12.25  12.81  10.85 | **13.09 ± 0.5**  12.62  14.33  13.33 | **14.68 ± 0.35**  15.19  14.00  14.85 | **15.66 ± 0.8**  14.06  16.34  16.59 | **15.78 ± 0.7**  15.88  14.52  16.95 |
| ***mascRNA*** | **19.2 ± 0.3**  19.51  19.42  18.66 | **18.59 ± 0.27**  18.11  19.07  18.6 | **18.08 ± 0.38**  18.6  18.29  17.35 | **18.06 ± 0.37**  17.45  18.74  18 | **17.91 ± 0.06**  17.81  17.9  18.01 | **9.39 ± 0.26**  9.66  9.63  8.88 | **17.44 ± 0.12**  17.68  17.38  17.27 | **12.74 ± 0.68**  12.25  11.61  12.04 | **15.74 ± 0.05**  15.72  15.86  15.69 |
| ***H19*** | **10.2 ± 0.45**  9.34  10.38  10.88 | **14.82 ± 0.29**  14.75  14.35  15.36 | **12.78 ± 0.32**  13.18  13.03  12.15 | **8.64 ± 0.39**  9.18  8.86  7.89 | **11.58 ± 0.43**  12.19  10.75  11.81 | **12.36 ± 0.34**  11.90  13.02  12.16 | **15.49 ± 0.31**  15.68  15.91  14.88 | **12.93 ± 0.63**  12.84  14.08  11.89 | **14.66 ± 0.15**  14.39  14.68  14.91 |
| ***SRA*** | **11.25 ± 0.46**  11.70  11.72  10.34 | **8.99 ± 0.84**  8.91  10.49  7.58 | **11.75 ± 0.33**  11.20  12.35  11.72 | **8.87 ± 0.69**  8.98  7.63  10.01 | **8.40 ± 1.15**  6.16  9.95  9.10 | **11.29 ± 0.13**  11.27  11.54  11.08 | **10.98 ± 0.47**  10.77  11.89  10.30 | **6.85 ± 0.21**  7.08  7.04  6.43 | **7.42 ± 0.57**  7.19  8.51  6.57 |
| ***MEG3*** | **25.36 ± 0.42**  24.82  26.18  25.08 | **24.94 ± 0.19**  24.71  24.78  25.32 | **25.17 ± 0.42**  25.93  25.08  24.5 | **24.1 ± 0.26**  23.59  24.34  24.38 | **22.82 ± 0.19**  22.52  22.76  23.17 | **20.89 ± 0.93**  19.83  20.74  20.09 | **22.47 ± 0.17**  22.16  22.74  22.52 | **18.17 ± 0.36**  17.51  18.24  18.76 | **13.42 ± 0.22**  13.24  13.85  13.17 |
| ***Neat1v1*** | **16.98 ± 0.49**  17.69  17.21  16.04 | **18.35 ± 0.41**  17.54  18.7  18.82 | **19.27 ± 0.7**  20.07  19.88  17.87 | **16.31 ± 0.08**  16.19  16.27  16.46 | **19.84 ± 0.3**  19.93  20.31  19.27 | **12.96 ± 1.3**  13.02  15.18  10.68 | **18.48 ± 0.28**  18.45  18.97  18.01 | **12.07 ± 0.14**  12.26  11.79  12.15 | **14.6 ± 0.37**  13.87  14.82  15.11 |
| ***Malat1*** | **14.37 ± 0.13**  14.3  14.18  14.63 | **14.57 ± 0.36**  13.85  14.9  14.97 | **13.3 ± 0.3**  13.83  13.26  12.81 | **13,91 ± 0.08**  13.94  14.03  13.76 | **13.92 ± 0.24**  14.24  14.07  13.45 | **10.71 ± 0.53**  10.99  11.45  9.68 | **12.98 ± 0.13**  12.89  13.23  12.81 | **11.6 ± 0.34**  12.03  11.83  10.94 | **13.54 ± 0.53**  12.88  13.17  14.58 |

**Table S12. Primers and probes used in the study.**

| **lncRNA** | **Primers** | **Temp. for qPCR and amplicon size** | **Ref.** |
| --- | --- | --- | --- |
| *18S* | CTTAGAGGGACAAGTGGCG  ACGCTGAGCCAGTCAGTGTA | 47°C  106 bp | [74] |
| *H19* | TACCCCGGGATGACTTCATC  TATCTCCGGGACTCCAAACC | 47°C  185 bp | [75] |
| *SRA* | GGCGGGCTGGTGGTACTCG GCGTCGGCTGATATCATCACATACC | 55°C  434 bp | [76] |
| *ppp1r1b* | GCATCTGAGCAGCTGTGCAGCA  CCTCCTCATCATCCTCCTGTGGGT | 55°C  155 bp | [47] |
| *yylncT* | CACAACACAAACTGGCTGGT  CTGCCCTTTCCCACTGAATA | 49°C  221 bp | [28] |
| *linc-YY1* | AGTTACAGGGAAGTTTGGGCTAC  AGGCAAAGGACGGCTGTGAG | 52°C  219 bp | [46] |
| *lncMyoD* | GATGTGAATCCCGGTTCTGC  GGAGTAAGATGGGTGTGGCT | 47°C  152 bp | [54] |
| *Sirt1-AS* | AATCCAGTCATTAAACGGTCTACAA  TAGGACCATTACTGCCAGAGGA | 47°C  265 bp | [34] |
| *Mrhl* | CTGCACACACACAAACACACA  AGGGTTCTCTGTCCTGACCT | 49°C  123 bp | [29] |
| *mascRNA* | GACGCTGGTGGCTGGCACT  TGGAGACACCGCAGGGAC | 54°C  57 bp | [33] |
| *Neat1v1* | TTGGGACAGTGGACGTGTGG  TCAAGTGCCAGCAGACAGCA | 60°C  106 bp | [77] |
| *Malat1* | CGGCTTTGGTTCACAGTCAC ACACAAGGCCACAGCCAACT | 63°C  138 bp | [77] |
| *MEG3* | CGAGGACTTCACGCACAA  ATTCCAGATGATGGCTTTG | 48°C  62 bp | [78] |
| **Gene** | **TaqMan® Gene Expression Assay probes** | **Amplicon lenght** | **Source** |
| *Actb* | Mm01205647_g1 | 72 bp | Thermo Fisher Scientific |
| *T* | Mm01318252_m1 | 57 bp |  |
| *Pdgfrα* | Mm00440701_m1 | 66 bp |  |
| *Kdr* | Mm01222421_m1 | 64 bp |  |
| *Myf5* | Mm00435125_m1 | 71 bp |  |
| *Myhc3* | Mm01332463_m1 | 96 bp |  |
| *Myhc7* | Mm01319006_g1 | 66 bp |  |
| *Myhc2* | Mm01332564_m1 | 103 bp |  |
